# Specificity landscapes of 40 R2R3‐MYBs reveal how paralogs target different *cis*‐elements by homodimeric binding

**DOI:** 10.1002/imt2.70009

**Published:** 2025-03-05

**Authors:** Tian Li, Hao Chen, Nana Ma, Dingkun Jiang, Jiacheng Wu, Xinfeng Zhang, Hao Li, Jiaqing Su, Piaojuan Chen, Qing Liu, Yuefeng Guan, Xiaoyue Zhu, Juncheng Lin, Jilin Zhang, Qin Wang, Honghong Guo, Fangjie Zhu

**Affiliations:** ^1^ Haixia Institute of Science and Technology, National Engineering Research Center of JUNCAO, College of JUNCAO Science and Ecology, Fujian Provincial Key Laboratory of Haixia Applied Plant Systems Biology Fujian Agriculture and Forestry University Fuzhou China; ^2^ College of Life Science Fujian Agriculture and Forestry University Fuzhou China; ^3^ College of Resources and Environment Fujian Agriculture and Forestry University Fuzhou China; ^4^ State Key Laboratory for Conservation and Utilization of Subtropical Agro‐Bioresources South China Agricultural University Guangzhou China; ^5^ Department of Biomedical Sciences City University of Hong Kong Hong Kong China; ^6^ Tung Biomedical Sciences Centre City University of Hong Kong Hong Kong China; ^7^ Department of Precision Diagnostic and Therapeutic Technology The City University of Hong Kong Shenzhen Futian Research Institute Shenzhen China

**Keywords:** *cis*‐elements, DNA binding specificity, homodimerization, MYB family transcription factors, paralogs, regulatory noncoding genome, transcriptional regulation

## Abstract

Paralogous transcription factors (TFs) frequently recognize highly similar DNA motifs. Homodimerization can help distinguish them according to their different dimeric configurations. Here, by studying R2R3‐MYB TFs, we show that homodimerization can also directly change the recognized DNA motifs to distinguish between similar TFs. By high‐throughput SELEX, we profiled the specificity landscape for 40 R2R3‐MYBs of subfamily VIII and curated 833 motif models. The dimeric models show that homodimeric binding has evoked specificity changes for AtMYBs. Focusing on AtMYB2 as an example, we show that homodimerization has modified its specificity and allowed it to recognize additional *cis‐*regulatory sequences that are different from the closely related CCWAA‐box AtMYBs and are unique among all AtMYBs. Genomic sites described by the modified dimeric specificities of AtMYB2 are conserved in evolution and involved in AtMYB2‐specific transcriptional activation. Collectively, this study provides rich data on sequence preferences of VIII R2R3‐MYBs and suggests an alternative mechanism that guides closely related TFs to respective *cis‐*regulatory sites.

## Highlights

- A homodimerization‐based mechanism enables eukaryotes to distinguish paralogous transcription factors.
- The largest plant SELEX data set illustrates the sequence preferences of 40 VIII R2R3‐MYBs.
- The high specificity AtMYBs were discovered and named CCWAA‐box.

## Graphical abstract

Closely related transcription factor (TF) paralogs are facing the “specificity paradox”—they share similar binding motifs, but their *cis‐*regulatory targets and physiological roles can be different. By applying high‐throughput SELEX to 40 R2R3‐MYB TFs, this study currently generates the largest data set illustrating the homodimeric specificities of plant TFs, while also reveals a yet unrecognized mechanism to solve the “specificity paradox”—a TF's binding specificity can change drastically upon homodimerization, and become unique across the whole family.

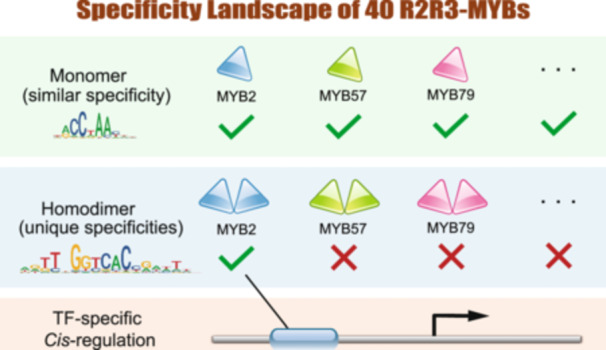

## INTRODUCTION

The spatial and temporal expression profile of genes is largely determined by the transcription factors (TFs) that bind to specific DNA sequences and activate the target genes. Specificity describes the degree of discrimination when a TF binds different DNA sequences, that is, how “picky” the TF is upon DNA binding. The specificity of a TF is typically measured by the information content (IC) of the TF's binding motif. By recognizing long binding motifs (~23 bits of IC) [1, 2, 3, 4], prokaryotic TFs have sufficient specificity to define ~10 targets in their ~10^6^ bp genome. In contrast, eukaryotic TFs are facing a specificity paradox. This is because they recognize much shorter DNA motifs (~12.1 bits of IC) [1, 5, 6, 7] that would define ~10^5^ binding sites in the ~10^9^ bp genome, but practically, a eukaryotic TF has to specifically regulate only 10–1000 genes [8, 9]. Moreover, the specificity paradox also applies to TFs of the same family. Whereas closely related TFs tend to bind highly similar sequences, their binding sites and target genes, as well as their physiological functions, can be distinct from each other [10, 11]. While epigenetic modifications [12, 13], chromatin contexts [11, 14], and TFs' DNA shape preference [15, 16, 17, 18, 19] have partially resolved this paradox by restricting the suitable target sites, dimerization of TFs also represents a straightforward solution that increases both the specificity [20, 21] and affinity [22] of eukaryotic TFs.

Homodimerization occurs between monomers of the same TF. It is known to help discriminate between closely related paralogous TFs because the dimeric configurations (relative spacing and orientation) can be unique for each TF. For example, the homodimeric configuration has contributed to distinguishing between the closely related animal TFs of families like ETS, T‐box, forkhead, RHD, and homeodomain [23]. In plants, the importance of dimeric configuration was demonstrated for the auxin response factors (ARFs)‐family TFs. Although ARFs within the same clade share a similar sequence preference for monomers [24], their homodimers prefer different relative orientations and spacings [25]. Moreover, changes to homodimeric configurations also distinguish the same TF in different species in evolution [26]. Despite these findings, whether homodimerization affects the sequence specificity of TFs, and whether such effect is shared by all closely related TFs or can be specific to individual TFs, is yet to be explored.

MYB represents one of the largest families of plant TFs in higher plants [27, 28]. The size expansion of MYB TFs is mainly attributed to the rapid expansion of the R2R3‐MYBs during green plant evolution [29, 30], and especially to the subfamily VIII R2R3‐MYBs that emerged in the common ancestor of Zygnematophyceae and land plants [30]. The sequences of the R2R3‐MYB TFs feature two highly conserved MYB repeats that bind to DNA. Members of subfamily VIII R2R3‐MYB TFs regulate numerous plant‐specific physiological processes, such as the synthesis of secondary metabolites [31, 32], deposition of cell walls [33], responses to biotic and abiotic stresses [34, 35], formation of cuticle and trichomes [36], and development of stamens [37]. In stark contrast to the diverse physiological functions of VIII R2R3‐MYBs, the publicly available high‐throughput data of DNA affinity purification sequencing (DAP‐seq) and protein binding microarray (PBM) [7, 13, 38, 39, 40] indicate that their monomers bind to highly similar and short DNA motifs [30, 40] (Figure S1A). Therefore, how VIII R2R3‐MYB TFs solve the “specificity paradox” to target different downstream pathways is of biochemical interest and physiological importance.

Subfamily VIII AtMYBs often dimerize in transcriptional regulation [41]. Homodimers have also been extensively reported for VIII AtMYBs (e.g., AtMYB21, AtMYB24 [42], AtMYB3 [43], and AtMYB82 [44]). However, DAP‐seq and PBM libraries of VIII R2R3‐MYB TFs have provided limited insights into their dimeric binding. This is because for PBM, the length of the randomized region on the probes is ~10 bp, which, in general, is not adequate to accommodate the dimeric binding modes. For DAP‐seq, the complexity of the input library is restricted by the size of the genome. DAP‐seq usually results in thousands of peaks; the limited number of sequences from DAP‐seq peaks makes it difficult to derive dimeric binding models of TFs (e.g., very few dimeric models were found for VIII AtMYBs; Data S1) because these sequences represent an undersampling of the high‐information‐content dimeric binding modes.

Here, we systematically analyzed with high‐throughput systematic evolution of ligands by exponential enrichment (HT‐SELEX) the binding preferences of 40 R2R3‐MYBs of subfamily VIII. The complexity (10^12^ input sequences) and ligand length (101‐bp randomized region) of the SELEX library allow a comprehensive characterization of both the monomeric and dimeric specificities (Data S2). The monomeric specificities revealed that a group of “CCWAA‐box” AtMYBs feature a distinct specificity and tend to be more selective than other VIII AtMYBs. The homodimeric specificities suggest that when two molecules of VIII AtMYBs bind closely to each other, the binding specificity of the half‐sites can change drastically. This has allowed AtMYB2 to recognize *cis‐*regulatory codes that are unique among all AtMYBs, thereby representing an alternative solution to the “specificity paradox” faced by paralogous eukaryotic TFs.

## RESULTS

### Specificity landscapes reveal a high selectivity of CCWAA‐box MYBs

To profile the landscape of DNA‐binding specificity of VIII R2R3‐MYBs, 3–5 cycles of high‐throughput SELEX enrichment were carried out. The enrichment suggests that SELEX has evolved high‐affinity ligands (Figure S2A–C). Replicates of SELEX libraries agree well with each other (Figure S2C). The mutual‐information‐based analyses [45] (Figure S2D) also confirmed the enrichment of TF‐preferred DNA sequences in the SELEX libraries.

**Figure 1:**
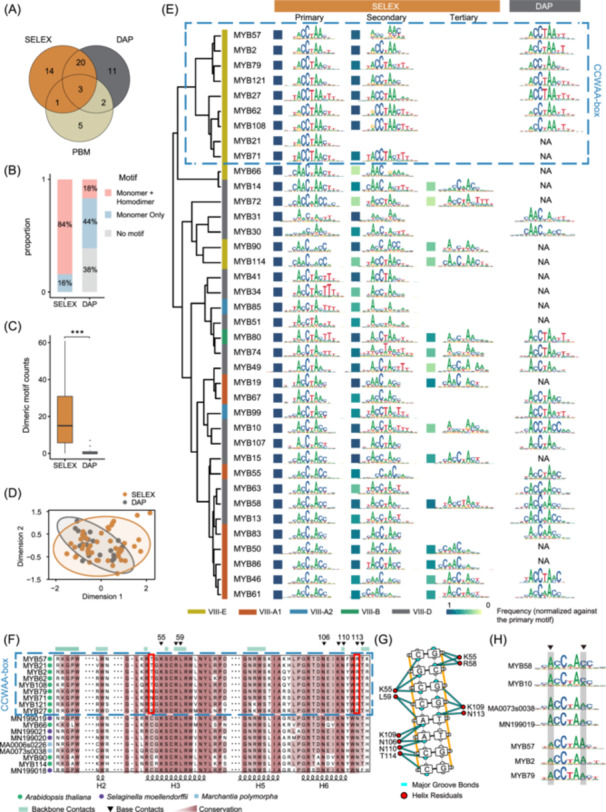
The landscape of binding specificity of VIII R2R3‐MYBs. (A) Comparison of SELEX and previous datasets. (B) Successful rates of motif discovery. The fractions of libraries that yield TF motifs (with Autoseed) are shown. (C) Numbers of dimeric motifs derived from individual libraries. ***p < 0.001 in *t*‐test. (D) The specificity space of monomeric motifs. Multidimensional scaling results of the monomeric motifs. The normal data ellipses (95%) were also shown to facilitate comparison. (E) The monomeric binding preferences of VIII R2R3‐AtMYBs. Motif logos are discovered from the HT‐SELEX libraries, and also from the published DAP‐seq libraries [13]. PBM library is used for AtMYB46. The color of the bar to the left of the TF names indicates the clades of VIII R2R3‐AtMYBs [30]. The color of squares to the left of the motifs represents its relative enrichment compared to the primary motifs. The CCWAA‐box AtMYBs were boxed with blue dashes. (F) Sequence alignment of the DBDs of VIII‐E R2R3‐MYBs. The VIII‐E AtMYBs studied in this work are aligned with all VIII‐E MYBs in *M. polymorpha* and *S. moellendorffii*. DNA‐contacting residuals are annotated based on the structure of AtMYB66 (PDB: 6KKS [46], also included in alignment). The two mutations at positions 53 and 113 (red boxes) likely accounted for the unique specificity of the CCWAA‐box AtMYBs (blue box). (G) Contacts between AtMYB66 and DNA bases. PDB: 6KKS visualized by DNAproDB [47]. (H) Binding specificities of ancestral VIII‐E R2R3‐MYBs. SELEX motifs of MA0073s0038 and MN199019. Note that their motifs are more similar to most VIII AtMYBs (e.g., AtMYB10, 58) and are different from CCWAA‐box AtMYBs (e.g., AtMYB57, 2, 79; positions with large discrepancies are gray‐shaded).

The SELEX libraries include 38 Arabidopsis VIII MYBs (AtMYBs), 14 of which were not covered by previous PBM and DAP‐seq datasets (Figure 1A). However, also for the overlapping datasets, the SELEX libraries can offer more binding models (Figure 1B), especially the dimeric binding models (Figure 1B,C). Moreover, the monomeric motifs generated by SELEX cover a larger specificity space compared to the DAP‐seq motifs (Figure 1D). This presumably is a consequence of the nonrandom nature of the DAP‐seq input sequences—the motifs from DAP‐seq reflect not only the biochemical affinity of the *cis*‐elements but also their genomic prevalence. Therefore, the SELEX data set complements the previous PBM/DAP datasets.

Next, we examined the monomeric binding preferences of the VIII AtMYBs and clustered them according to their primary motifs (Figure 1E). *De novo* motif discovery reveals that the examined AtMYBs enriched primary, secondary, and tertiary motifs (Figure 1E). Consistent with the PBM studies [7], when multiple monomeric motifs exist for the same AtMYB, they are usually quite similar. This is different from TFs of bZIP [20], C2C2‐Dof, and DREB [7] families, for which the secondary motif could significantly diverge from the primary motif. In general, the monomeric motifs derived from SELEX agree well with the DAP‐seq motifs (Figure 1E and Figure S1A). Although with weaker enrichments, SELEX is capable of discovering secondary/tertiary motifs that are usually not detected in DAP‐seq.

While the majority of the VIII AtMYBs prefer to bind DNA sequences summarized by “AMCWAMY” (W: A/T; M: A/C; Y: C/T), many AtMYBs of the VIII‐E clade [30] prefer to bind “RCCWAAY” (CCWAA‐box) as the consensus (blue box; Figure 1E) and feature a higher specificity than other AtMYBs (Figure 1E), that is, motifs of CCWAA‐box AtMYBs are with higher ICs (Figure S1C). The CCWAA‐box AtMYBs likely originated from a common ancestor (within the same monophyletic clade; Figure S1B). To find potential amino acids that account for their distinct specificity, we next aligned the sequences of the VIII AtMYBs studied in this work, together with all the VIII MYBs in *M. polymorpha* and *S. moellendorffii*, whereby the VIII subfamily of MYBs has not expanded (Figure 1F and Figure S2E). We focused on the amino acid sites that are both conserved in evolution (thus higher probability to involve in DNA binding) and differ between CCWAA‐box AtMYBs and other MYBs. We found that mutations in positions 53 and 113 (red frames in Figure 1F and Figure S2E) presumably explained the distinct specificity. The base contacts by asparagine at position 113 have also been validated by the structural data of AtMYB66 [46] (Figure 1F,G), and by the AlphaFold structure of AtMYB2 (with R113; Figure S2F). Further examination of the sequences in *M. polymorpha* and *S. moellendorffii* suggests that consistent with the majority of VIII AtMYBs, ancestors of VIII‐E clade MYBs (Figure 1F) and other clades of VIII MYBs (Figure S2E) also contain C, N at positions 53, 113. In agreement with the sequence analyses, we performed SELEX for MA0073s0038 (*M. polymorpha*) and MN199019 (*S. moellendorffii*), and found that they bind to “ACCWAMM” (Figure 1H), which is more similar to the “AMCWAMY” (most other AtMYBs, e.g., AtMYB58/10 in Figure 1H) than the “RCCWAAY” (CCWAA‐box AtMYBs). Therefore, the distinct specificity of CCWAA‐box VIII‐E AtMYBs could have originated from their unique AA identities at positions 53 and 113.

### Modified specificity of closely spaced AtMYB2 homodimers

**Figure 2:**
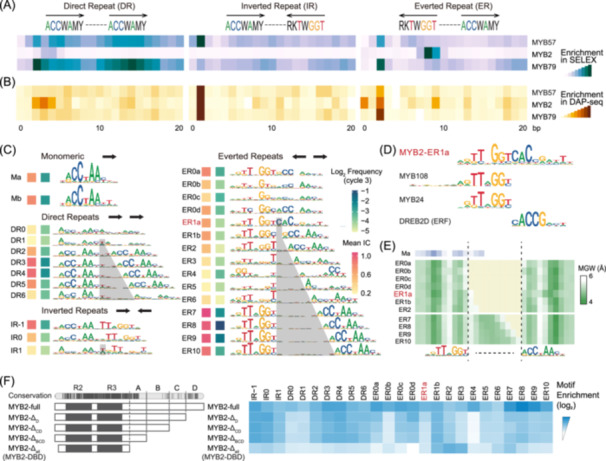
Modified specificities of closely spaced AtMYB2 homodimers. (A) AtMYBs with similar monomeric motifs have different homodimeric preferences. Enrichments of dimeric AtMYB CREs with different spacings and three relative orientations in SELEX libraries. Two “ACCWAMY” strings are concatenated with the annotated spacing (*x*‐axis) and assessed for enrichment. (B) Homodimeric preferences of genomic AtMYB CREs. Enrichments of dimeric AtMYB CREs with different spacings and three relative orientations in DAP‐seq peaks. (C) Monomeric and homodimeric motifs of AtMYB2. *De novo* discovered motifs in the AtMYB2 SELEX library, with their information content (red squares) and enrichment (green squares) indicated to the left. Dimeric motifs were named according to their relative orientations and spacings. The spacing regions are colored gray (half‐site: ACCWAAY). Note that half‐sites in ER1s and ER0s are less similar to the monomeric motifs, especially for ER1a (red). (D) ER1a is dissimilar to reported plant motifs. The ER1a motif was compared to all reported plant motifs [6, 48, 49], and the three most similar known motifs are shown. (E) Distinct DNA‐shape preference of ER1a. Average minor groove widths are illustrated for each position of the sequences harboring respective motifs of AtMYB2. Positions are aligned with additional gaps (the light yellow zone between the vertical dashes) to separate the two half‐sites of ER0–2. Note that the profile is similar for half‐sites of all motifs except for ER1a. (F) Non‐DBD region affects AtMYB2 dimeric preferences. SELEXs with progressively truncated AtMYB2 in the non‐DBD region reveal that segments A and D determine the dimeric configurations of AtMYB2 because the dimeric preference changes dramatically when truncating A and D, but only slightly when truncating B and C. Accordingly, A and D are also evolutionarily more conserved (Figure S3H). The degree of conservation is also summarized across AtMYB2.

The closely related AtMYBs are further discriminated by their homodimeric configurations (Figure 2A and Figure S3A). For example, the CCWAA‐box AtMYBs 2, 57, and 79 were neighbors in the clustering and feature highly similar motifs (Figure 1E); however, their preferred homodimeric spacings and orientations are distinct from each other (Figure 2A). The most preferred dimeric orientation for AtMYB2 is everted repeats (ER), while inverted repeats (IR) are most preferred by AtMYB57, and direct repeats (DR) by AtMYB79 (Figure 2A). The AtMYBs also prefer different dimeric spacings. For the DR configurations, while AtMYB2 specifically binds DR3 and DR4, AtMYBs 57 and 79 bind to almost all DR modes (Figure 2A). Although *de novo* discovery can hardly find dimeric motifs from DAP‐seq libraries (Figure 1B, Data S1), the enrichment analysis is still possible and gives insights into configurational preferences of homodimeric *cis‐*regulatory elements (CREs). Next, we generated DAP‐seq libraries for AtMYB2/57/79 and analyzed the homodimeric configurations of their genomic CREs (Figure 2B and Figure S3B). Consistent with previous analyses on dimeric WRKY CREs [50], the homodimeric configurations of the AtMYBs in DAP‐seq resemble those in SELEX but also bear discrepancies, likely because thermodynamic selection and drifts both contributed to shaping the *cis‐*regulatory codes in evolution [50].

Because the enrichment analysis with consensus cannot determine whether the sequence preferences of AtMYBs have changed upon homodimerization, we next systematically discovered and curated the homodimeric motifs for AtMYBs (Figure 2C, all motifs visualized in Data S2, data in Tables S4–S41). In total, we report 743 homodimeric motifs of AtMYBs in this study. The dimeric motifs were named according to the relative orientations (DR, IR, and ER) and spacings between the monomers (Figure 2C). In public databases [5, 6, 48, 49], the majority of the reported plant motifs for AtMYBs were monomeric (Figure S1A). The identified dimeric motifs could add extra insights into the combinatorial regulatory codes of AtMYBs. Notably, we observed that AtMYBs can change their DNA‐binding specificity when two monomers bind closely to each other (Figure 2C and Figure S3C), that is, the half‐sites of the dimeric motifs diversify from the monomeric motif. Such specificity change is particularly prominent for AtMYB2 (Figure 2C).

For AtMYB2, the discovered dimeric motifs encompass all relative orientations of the monomers (Figure 2C), with ER8 being the strongest dimeric mode. The motif models more enriched in SELEX (green squares in Figure 2C) generally also feature higher information contents (IC, red squares in Figure 2C). The closely spaced homodimers in the ER conformation are associated with high ICs (thus sufficient affinity and specificity) and represent reliable binding modes (ER0s and ER1s). In contrast with the distantly spaced ER modes (ER7–10; Figure 2C) whereby the half‐sites are almost the same as the monomeric motif Ma, the specificity has changed upon proximal homodimerization (ER0s and ER1s) and is especially drastic for ER1a (name in red; Figure 2C). The half‐site on the right of ER1a is completely different from the monomeric modes Ma and Mb. ER0s and ER1s also align poorly with previously reported plant motifs (Figure 2D and Figure S3D), suggesting that these dimeric modes were not reported before. The two binding events in all the identified dimeric modes are cooperative (Figure S3E).

In addition to individual nucleotides, the shape of local DNA sequences also contributes to the specificity of TF binding [15, 16, 17] and aids in discriminating binding sites of closely related TFs [18, 19, 51]. We examined the DNA‐shape parameters for the SELEX reads that matched each ER dimeric motif with a high IC, and found that the half‐sites of most dimeric modes feature DNA‐shape patterns that resemble the shapes of the monomeric AtMYB2 motif (Figure 2E and Figure S3F). A narrowed minor grove is observed for the spacing regions between the two half‐sites (Figure 2E). However, ER1a differs from other ER modes for most of the shape parameters, for example, the minor grove width (Figure 2E), buckle, rise, shift, and opening (Figure S3F). Therefore, proximal homodimeric binding not only induces changes to the nucleotide specificity of AtMYB2 but also to its DNA‐shape specificity.

The non‐DBD (DNA binding domain) region was shown to affect the dimerization and DNA‐binding specificity of TFs [52, 53]. Similarly, we found that the non‐DBD region of AtMYB2 is critical for its dimerization (MYB2‐full vs. MYB2‐Δ_all_; Figure 2F). Consistently, the AlphaFold structures of AtMYB2‐ER8 show that the non‐DBD region is indispensable for the contacts between the two AtMYB2 monomers (Figure S3G). In a higher resolution, the non‐DBD segments D and A are the major determinants of the dimeric configurations. Truncation of segment D has decreased the distantly spaced dimeric modes (ER7–9) of AtMYB2 (MYB2‐full vs. MYB2‐Δ_D_; Figure 2F), and truncation of segment A has further abolished almost all of the dimeric modes (DR3/4, IR0/‐1, and ER0/1s) while selectively enhanced ER2 and ER3 (MYB2‐Δ_BCD_ vs. MYB2‐Δ_all_; Figure 2F). In contrast, only slight changes were observed when truncating segments B (MYB2‐Δ_CD_ vs. MYB2‐Δ_BCD_; Figure 2F) and C (MYB2‐Δ_D_ vs. MYB2‐Δ_CD_; Figure 2F). Accordingly, segments A and D are also more conserved than B and C in evolution (Figure 2F and Table S3), and segment D likely also represents the activation domain (Figure S3H). These results underscore the importance of the non‐DBD region in shaping the dimeric binding specificities.

Because AtMYB2 drastically changes its specificity when binding as closely spaced ER homodimers, we next focused on ER0s and ER1s (especially ER1a) of AtMYB2 to explore their uniqueness, biochemical activity, and transcriptional regulatory relevance.

### The modified specificities are unique to AtMYB2

**Figure 3:**
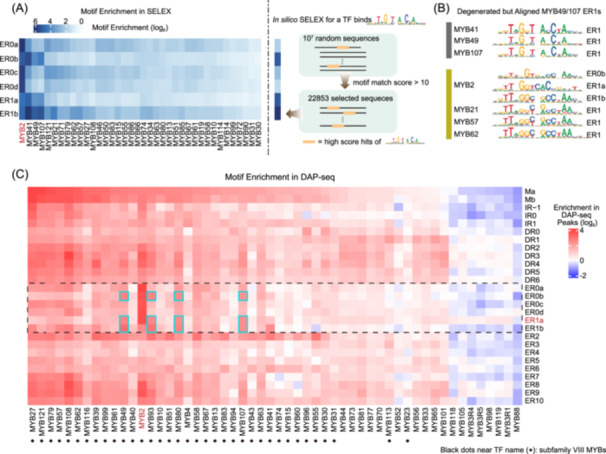
The modified specificities are unique to AtMYB2. (A) AtMYB2 enriches unique ER0s and ER1s in SELEX. Enrichments of AtMYB2 ER0s and ER1s in SELEX libraries of AtMYBs (left). For a virtual TF that binds only the ER1 of AtMYB41/49/107 (right), its in silico SELEX also strongly enriched ER0b, 1a, 1b of AtMYB2, suggesting that the observed enrichments for AtMYB41/49/107 in the left panel are false positives and that AtMYB2 has unique ER0 and ER1 modes, except that ER1b is also bound by AtMYB21/57/62. (B) Degenerated AtMYB41/49/107 ER1s are aligned with AtMYB2 motifs. The ER1s of MYB41, 49, 107, 21, 57, and 62 are compared to ER0b, 1a, 1b of AtMYB2. The bar color indicates the clades of the VIII AtMYBs according to Figure 1E. (C) AtMYB2 enriches unique ER0s and ER1s in DAP‐seq peaks. Enrichments of AtMYB2 motifs are surveyed for DAP‐seq peaks. Note that ER0s and ER1s of AtMYB2 (dashed box) are uniquely enriched for AtMYB2. Similar to the observation for SELEX (A, B), the enrichments of ER0b, 1a, 1b for AtMYB49, 107, 93, and 80 (cyan boxes) are presumably from their degenerated ER1 motifs. Note that the specificity‐changed ER0/1s are only enriched for AtMYB2 here but not in (Figure 2B), whereby the use of fixed strings cannot capture the specificity changes.

To examine whether the modified specificity of ER0s and ER1s are unique to AtMYB2, we explored the enrichments of ER0s and ER1s in SELEX libraries of all AtMYBs (Figure 3A). Although ER0s and ER1s were not detected for the majority of the other AtMYBs, we did observe that AtMYB41/49/107 enriched ER0b, 1a, and 1b. Because motif enrichment analysis is subject to false positives, we next inspected the *de novo* motifs from SELEX and found that AtMYB41/49/107 libraries did not contain the exact ER0b, 1a, and 1b of AtMYB2 (Figure 3B), but instead enriched degenerated ER1s that represent a larger set of preferred DNA sequences. The positions with high IC in the ER1s of AtMYB41/49/107 align with the ER0b, 1a, and 1b of AtMYB2 (Figure 3B); we thus suspect it is the presence of these ER1 modes that have resulted in the observed motif enrichments of ER0b, 1a, and 1b for AtMYB41/49/107 (Figure 3A). To validate this hypothesis, we generated an *in silico* SELEX library (Figure 3A) that enriches only the ER1 of AtMYB49 and used the library for enrichment analysis of AtMYB2's motifs. As expected, the simulated SELEX library also enriched the ER0b, 1a, and 1b of AtMYB2. Therefore, among the examined VIII AtMYBs, the observed ER0s and ER1s are unique to AtMYB2, with the exception that a motif similar to AtMYB2's ER1b is indeed discovered from libraries of AtMYB21/57/62 (Figure 3B).

To further validate the uniqueness of the ER0s and ER1s of AtMYB2, we exploited the highly abundant DAP‐seq datasets both for AtMYBs [13] and MYBs of other plants [54, 55, 56, 57, 58]; they also support enrichment analysis of available dimeric motifs. We examined the enrichment of AtMYB2 motifs within DAP‐seq peaks (Figure 3C and Figure S4). The results show that among the 54 DAP‐seq libraries of AtMYBs, almost all subfamily VIII R2R3‐AtMYBs (dots aside from TF name; Figure 3C) have enriched AtMYB2's monomeric motifs (Ma/Mb). This is in line with the similarity between monomeric motifs across the VIII R2R3‐AtMYBs (Figure S1A). AtMYB2's dimeric modes ER7–9 and DR2–4 also enriched in ~1/2 of the other VIII R2R3‐MYBs (Figure 3C). Among the homodimeric modes with modified specificities (ER0s and ER1s of AtMYB2; boxed with the dashed line in Figure 3C), ER0s and ER1a are uniquely and strongly enriched for AtMYB2, suggesting that these dimeric binding modes provide sufficient specificity to distinguish AtMYB2 from other AtMYBs. A few other AtMYBs also moderately enrich ER0s and ER1s, for example, the AtMYBs 49, 80, 93, and 107 (cyan boxes; Figure 3C). As already validated for AtMYB49 and 107 (Figure 3A,B), such enrichments presumably originate from sequences corresponding to more degenerated motifs.

Moreover, we analyzed the published DAP‐seq libraries of MYB‐family TFs for *Eucalyptus grandis*, *Oryza sativa*, *Vitis vinifera*, and *Camelina sativa*. Similar to AtMYBs (Figure 3C), many of these DAP‐seq libraries enriched the monomeric modes Ma/Mb, but strong enrichments of ER0s and ER1a were not observed (Figure S4), further confirming their uniqueness to AtMYB2.

### The modified specificities allow AtMYB2 to recognize additional genomic sites

**Figure 4:**
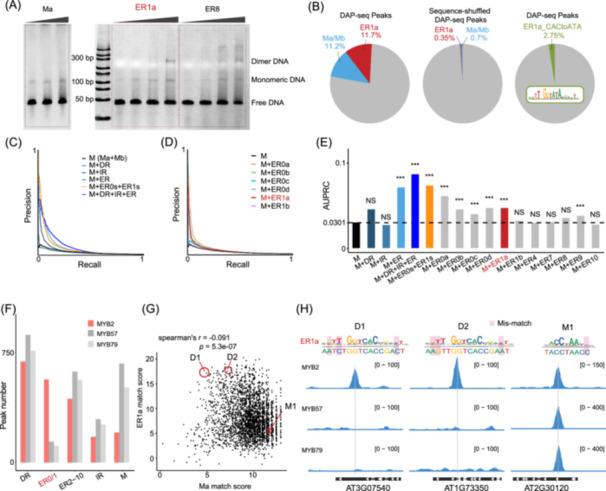
The modified specificities allow AtMYB2 to recognize additional genomic sites. (A) ER1a is functional for the dimeric binding of AtMYB2. Electrophoretic mobility shift assay of AtMYB2 binding to Ma, ER1a, and ER8 consensus. The concentrations of AtMYB2 are 18, 24, 36, 48 ng/µL from left to right (ER1a and ER8) and 24, 36, 48 ng/µL for Ma. (B) ER1a is enriched in genomic CREs of AtMYB2. Enrichment of ER1a in DAP‐seq peaks of AtMYB2 is comparable to the enrichment of the monomeric motifs Ma/Mb (left), whereas the shuffled DAP‐seq peaks show little enrichment of ER1a (middle). Mutations to CAC in the right half‐site of ER1a also decrease its enrichment (right). (C–E) ER0s and ER1s improve the accuracy of the prediction of AtMYB2 genomic CREs. Precision recall (PR) curves (C, D) and area under the curve (AUPRC) values (E) for motif‐based prediction of DAP‐seq peaks. Note that when combined with the monomeric motifs (M), the ER modes most significantly increased the prediction accuracy (E); further separating individual ER modes shows that ER1s and ER0s (D, E) with modified specificities have contributed most of the additional prediction power. Wilcoxon signed‐rank test (for paired precision values at each recall level) is performed to evaluate if adding the dimeric modes to M has significantly increased the predictive power. (F) ER0s and ER1s define unique targets of AtMYB2. ER0/1 sites are specifically enriched in DAP peaks of AtMYB2 but not in peaks of AtMYB57/79. This contrasts with IR, DR, and ER2–10. (G, H) The modified specificities of ER1a contribute to the affinity of AtMYB2 CREs. DAP‐seq peaks are scored with either the Ma and ER1a motifs of AtMYB2 (G). Peaks with low Ma scores but high ER1a scores were identified. Red circles highlight the three peaks that are exemplified in (H), whereby CRE sequences similar to ER1a but not to Ma (D1 and D2) specifically colocalize with the DAP peaks of AtMYB2 (CRE in peak indicated by gray), and the CRE sequence (M1) similar to Ma is associated with DAP peaks of AtMYB2/57/79, emphasizing the affinity and specificity contributions from the modified right half‐site of ER1a.

Because the specificity change is most drastic for ER1a, we next focused on ER1a as an example to validate its biochemical affinity to AtMYB2. With the recombinant AtMYB2 protein (Figure S5A,B), we performed EMSA with DNA ligands containing an ER1a consensus, together with ligands harboring Ma and ER8 for comparison (Figure 4A). We observed that all ligands are capable of binding a single molecule of AtMYB2. However, the shifted band according to dimeric binding of AtMYB2 is observed at high concentrations (36, 48 ng/µL) of AtMYB2 only for ER1a and ER8 ligands, but not for Ma ligands (Figure 4A), suggesting that although bearing a drastic specificity change, ER1a still represent a functional dimeric CRE of AtMYB2.

Moreover, we asked whether genomic sites of ER1a are recognized by AtMYB2. For this purpose, we further analyzed the DAP‐seq data of AtMYB2. Among the top 3000 peaks, there are 11.7% peaks contain one or more ER1a binding sites (Figure 4B). This fraction is comparable to that of the peaks containing monomeric AtMYB2 sites (11.2%) and is the highest among all AtMYB2's dimeric sites (Figure S5C). The number of ER1a sites diminishes when the peak sequences are shuffled (0.35%) or upon mutating the ER1a motif at the CAC nucleotides (2.75%; Figure 4B); therefore, the enrichment of ER1a is not due to mono‐nucleotide bias or the left half‐site of ER1a with unchanged specificity. These results suggest that genomic ER1a sites are recognized by AtMYB2 and contribute to the DAP‐seq signals.

Because DAP‐seq uses gDNA that is free of DNA‐associated proteins such as histones, the binding affinity of AtMYB2 to the genomic sites depends purely on the underlying DNA sequence. Therefore, we examined to which extent the DAP‐seq signals can be modeled by the curated SELEX motifs of AtMYB2. Based on the highest motif score of each sequence, a binary classification was performed to discriminate genomic regions into bound and unbound. Sequences in the DAP‐seq peaks are treated as bound, and the sequences outside the peaks are treated as unbound. The precision‐recall (PR) curves were generated by varying the threshold (Figure 4C,D and Figure S5D). The results show that combining the two monomeric motifs has relatively low predictive power (curve M; Figure 4C), with an AUPRC (area under the PR curve) of 0.0301. Adding DR motifs (but not IR) into the prediction can improve the accuracy (Figure 4C,E). However, the most significant improvement in prediction accuracy is observed when incorporating the ER motifs (AUPRC = 0.0863; Figure 4C,E). The additional predictive power of ERs mainly comes from the closely‐spaced ER motifs (ER0s and ER1a; Figure 4E and Figure S5E), but not from the ER motifs with large spacings (e.g., ER8; Figure 4E) despite their higher affinities (Figure 2C). This is consistent with the fact that drastic specificity changes primarily occur within half‐sites of ER0 and ER1 motifs (Figure 2C). Notably, adding ER1a alone to monomeric motifs has increased AUPRC from 0.0301 to 0.0471 (Figure 4D,E). Moreover, the ER0/1 s of AtMYB2 cannot improve the prediction of the DAP signals of the closely related AtMYB57/79 (Figure S5D). Consistently, we found that in the generated DAP libraries of AtMYB57/79, significantly fewer peaks contain sites of ER0/1 s compared to AtMYB2, whereas more peaks contain sites of Ma/Mb, DR, IR, and ER2–10 (Figure 4F). These comparative analyses suggest that ER0/1s are uniquely bound by AtMYB2.

To identify individual sites that contributed to ER1a's additional predictive power, we scored the DAP‐seq peaks of AtMYB2 with both ER1a and Ma (Figure 4G). The weak correlation and the existence of high ER1a score, and low Ma score peaks (e.g., the red circles in Figure 4G, coverage tracks, and binding site sequences exemplified in Figure 4H) suggest that the modified specificities in the right half‐site of ER1a also contributes to AtMYB2 binding, and help distinguish AtMYB2 from the closely related AtMYB57/79 (Figure 4H). Although both DAP‐seq and SELEX reflect the intrinsic biochemical affinity of a TF, discrepancies between the two libraries are observed for the enrichment of the AtMYB2 motifs (Figure S5F). Specifically, ER1a and ER0s are more enriched in DAP‐seq peaks than in SELEX reads (Figure S5F). Such discrepancy presumably reflects the preferred usage of *cis‐*regulatory codes in the genome resulting from evolutional drifts [50].

### The modified specificities of AtMYB2 are functional to activate transcription

**Figure 5:**
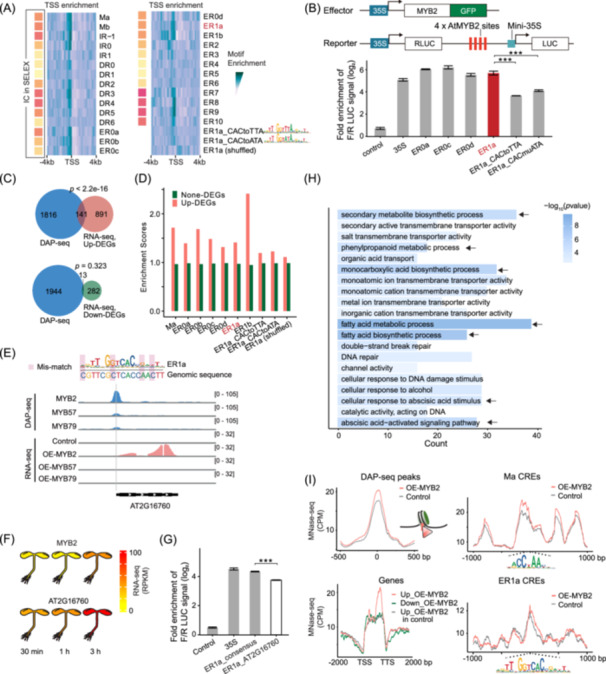
The modified specificities of AtMYB2 are functional to activate transcription. (A) ER0 and ER1 CREs enrich around promoters. Distributions of AtMYB2 CREs around transcription start sites (TSSs) of *A. thaliana* genes were evaluated by the density of motif matches. The yellow‐red squares indicate the information content of the motifs (same as in Figure 2C). Note that ER0s and ER1s are enriched around TSSs to an extent comparable with the monomeric motifs and that mutations to the right half‐site of ER1a (CACtoTTA/ATA) decreased its enrichment. (B) ER0s and ER1s of AtMYB2 activate transcription. (Top) Vectors used in the dual‐luciferase reporter assays. The consensus of each motif was repeated 4 times and inserted in front of a mini‐35S promoter. (Bottom) The reporter activity of ER0s and ER1a. ***p < 0.001 in *t*‐test. (C) Genes activated by AtMYB2 overlap with DNA affinity purification sequencing (DAP‐seq) peaks. DEGs were identified by comparing RNA‐seq data before and after overexpression of AtMYB2 in protoplasts. The DAP‐seq peaks of AtMYB2 significantly overlap with the upregulated DEGs but not with the downregulated DEGs, *p* values from Fisher's exact test. (D) Genes activated by AtMYB2 enrich modified dimeric specificities in promoters. Motif enrichments in the promoters of upregulated DEGs are compared to those in promoters of non‐DEGs. Note that mutations to the right half‐site of ER1a (CACtoTTA/ATA) decreased the enrichment. (E–G) AtMYB2 specifically binds to the promoter of AT2G16760 and activates its transcription. (E) The coverage tracks of RNA‐seq and DAP‐seq near the ER1a target AT2G16760. The sequence of the AtMYB2 CRE in the DAP‐seq peak (gray) is aligned with ER1a for comparison. (F) Expressions of both AtMYB2 and AT2G16760 are induced after ABA treatment of *Arabidopsis* seedlings (data from Expression Angler [59]). (G) Reporter assay comparing the activity of ER1a consensus to the AtMYB2 CRE sequence of AT2G16760. (H) The biological roles of AtMYB2. The GO enrichment of upregulated DEGs by AtMYB2 overexpression. Arrows indicate the GO terms related to ABA responses and metabolic pathways. (I) AtMYB2 binding is compatible with nucleosome occupancy. Nucleosome occupancy before and after overexpression of AtMYB2 in *Arabidopsis* protoplasts was measured by MNase‐seq. AtMYB2 has little effect on nucleosome occupancy before (gray line) and after (red line) overexpression near its binding sites altogether (DAP‐seq peaks) and its CREs of most individual motifs (Ma CREs and ER1a CREs). However, the DEGs upregulated by AtMYB2 overexpression show increased nucleosome occupancy near their TTSs (red line, Genes).

We further explored the roles of ER0s and ER1s of AtMYB2 in transcriptional regulation. First, we found that ER0s and ER1s are enriched around TSSs to an extent comparable with the monomeric motifs (Figure 5A). As a control, when we mutate the right half‐site of ER1a, TSS enrichment of the motif decreases (CACtoTTA and CACtoATA; Figure 5A). In general, SELEX motifs with higher IC contents (yellow‐red squares in Figure 5A) are also more enriched around the TSSs, suggesting that the high‐affinity binding modes identified by SELEX are more likely to be transcriptionally functional.

To experimentally validate whether ER0s and ER1s of AtMYB2 regulate transcription, we performed reporter assays using 4 × tandem repeats of these homodimeric motifs in *Arabidopsis* protoplasts (Figure 5B). In the presence of ectopic expression of AtMYB2, ER0s and ER1s can drive reporter gene expression to an extent higher than the constitutive 35S promoter and comparable to other high‐affinity dimeric modes (ER8, DR4, and IR0/‐1; Figure S6A). The activities of ER0/1s are also higher than two monomeric motifs (2 × Ma; Figure S6A). Mutating the right half‐site of ER1a decreases the promoter activity (Figure 5B); thus, it is the modified specificity of ER1a that allows dimeric binding of AtMYB2 and results in the observed high transcriptional regulatory activity. Consistent with the fact that AtMYB57 and 79 do not bind to ER0/1s (Figures 3A and 4F), when AtMYB57/79 is expressed, the transactivating capability of ER0a/ER1a is comparable or lower than two monomeric motifs (2 × Ma; Figure S6A), and much weaker than IR0, whereby half‐sites are with less specificity change. Therefore, the ER0/1s CREs are specifically functional for AtMYB2.

Next, we asked whether AtMYB2 can specifically activate downstream genes targeted by ER0s and ER1s. To this end, protoplasts from *Arabidopsis* leaves were measured by RNA‐seq before and after the ectopic overexpression (OE) of AtMYB2. In parallel, the OE of AtMYB57/79 was also performed for comparison. The OE has increased the transcript level of AtMYB2 by >100‐fold (Figure S6B) and resulted in 1349 differentially expressed genes (DEGs, |log_2_FoldChange | > 2, FDR < 0.05). We found that the upregulated genes significantly overlap with the DAP‐seq peaks of AtMYB2, whereas the downregulated genes do not (Figure 5C), supporting that AtMYB2 is a transcriptional activator rather than a repressor. Promoters of the upregulated genes have enriched 26 out of 27 AtMYB2 binding motifs identified in SELEX (Figure S6C), including the ER0s and ER1s (Figure 5D and S6D). Mutating the right half‐site of ER1a decreased the enrichment (CACtoTTA and CACtoATA; Figure 5A), suggesting that the changed specificity of ER1a has contributed to transcriptional activation. Multiple genes are upregulated by AtMYB2 through the ER1a CREs (Figure 5E and Figure S6E,F). For example, the most significantly upregulated ER1a target is AT2G16760 (Figure S6E), which encodes a calcium‐dependent phosphotriesterase superfamily protein. Accordingly, AtMYB2, but not AtMYB57/79, binds to the promoter of AT2G16760 and activates its transcription after overexpression (Figure 5E). Consistent with AtMYB2's role as an ABA‐responsive factor, the expression level of AT2G16760 also increases after ABA treatment (Figure 5F). However, the CRE targeting AT2G16760 is not a perfect ER1a site and harbors mismatches to the ER1a consensus (alignment in Figure 5E). We substituted all four mismatches back and observed an elevated expression in the reporter assay (Figure 5G), confirming the highest affinity and activity of ER1a consensus. Compared to DAP‐seq, the enrichment of AtMYB2 motifs in SELEX better correlates with the ability of these motifs to activate transcription (Figure S6G), potentially because DAP‐seq enrichment is additionally affected by the nonrandom genomic background aside from the affinity. Analyses of the DAP‐seq data of AtMYB2/57/79 have shown that ER1a CREs are specifically bound by AtMYB2 but not by AtMYB57/79 (Figure 4H); here, with the expression profiles, we further validated that genes harboring ER0/1 CREs in the promoter are specifically upregulated by OE of AtMYB2 but not OE of AtMYB57/79 (Figure S6H).

To explore the biological roles of AtMYB2, gene ontology (GO) analysis was performed for genes upregulated by AtMYB2 overexpression (Figure 5H). In agreement with the reported functions of AtMYB2 and MYB‐family TFs [34, 60, 61], biological processes of “abscisic acid‐activated signaling pathway” and those related to metabolism (arrows; Figure 5H) were enriched with the most significant *p*‐values. The genes upregulated by different dimeric modes have also enriched different GO terms (Figure S7A); the GO terms associated with ER0/1s could have contributed to the unique biological roles of AtMYB2.

In general, TF‐DNA bindings are incompatible with nucleosomes [45]. Upon binding to cognate CREs, TFs tend to evict or remodel nucleosomes and increase the nearby chromatin accessibility [62, 63]. However, when we measure the nucleosome occupancy by MNase‐seq for AtMYB2 binding sites, we found that AtMYB2 tends to bind nucleosomal DNA (DAP‐seq peaks; Figure 5I) and that OE of AtMYB2 has little effect on nucleosome occupancy near the AtMYB2 CREs. This is true both for the monomeric AtMYB2 CREs (Ma CREs; Figure 5I) and for the dimeric CREs that feature either the unchanged (ER8 CREs; Figure S7B) or changed specificities (ER1a CREs; Figure 5I). It is yet noteworthy that the genes upregulated by AtMYB2 OE have enriched nucleosomes at the end of the transcribing regions (Genes; Figure 5I). This enrichment of nucleosome is not observed for the downregulated genes, for the upregulated genes before AtMYB2 expression (Genes; Figure 5I), and for the non‐DEG genes (Figure S7C). Collectively, these observations suggest that DNA‐binding of AtMYB2 does not seem to interfere with nucleosome occupancy and that AtMYB2 is a potential “pioneer TF.” This is also supported by the observed short monomeric motifs of AtMYBs (Figure 1G), which may enable the shallow binding [63, 64] that minimizes the steric hindrance when TFs bind to nucleosomal DNA.

### The modified specificities of AtMYB2 are conserved in evolution

**Figure 6:**
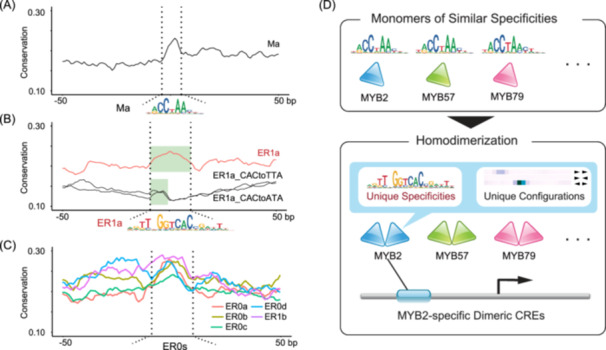
The modified specificities of AtMYB2 are conserved in evolution. (A–C) The conservation scores around AtMYB2 CREs. PhastCons scores (across 63 angiosperms) near promoter CREs corresponding to the AtMYB2 motifs Ma (A), ER1a (B), and ER0s and ER1b (C). Note that peak widths of the conservation scores agree with the widths of the SELEX motifs of AtMYB2 and that mutating the right half‐site of ER1a narrows its conservation peaks (CACtoTTA/ATA in B). (D) Homodimerization helps distinguish closely related TFs. CCWAA‐box VIII MYBs share highly similar monomeric motifs. However, upon homodimerization, their unique dimeric specificities and dimeric configurations (spacing and relative orientation) help discriminate between these AtMYBs, defining unique CREs of AtMYB2.

The existence of purifying selection, as indicated by a high level of sequence conservation, in general, is a reliable sign of functional CREs [65, 66]. We reasoned that if the homodimeric AtMYB2 CREs with modified specificities are physiologically functional, they should also be conserved during evolution. Therefore, we inspected the distribution of PhastCons scores (across 63 angiosperms [67]) around ER0/ER1 CREs located in the promoter region (Figure 6). As a positive control, we first validated that CREs of the monomeric mode (Ma) are more conserved than their flanks (Figure 6A). After that, we also interrogated the dimeric CREs with changed specificities and found that they also colocalized with peaks of conservation scores (Figure 6B,C). Notably, the widths of the conservation peaks are in accord with the width of the homodimeric CRE motifs. To further validate if the modified specificities in these motifs are functional, as an example, we mutated the CAC positions of the ER1a mode and visualized the nearby PhastCons scores for the putative CREs (Figure 6B). Accordingly, we found that the conservation peaks narrowed to span only the left half‐site, a width resembling the peak of the monomeric motif (Figure 6A). Taken together, the evolutional evidence further supports that the homodimeric motifs are not biochemical artifacts observed in SELEX, but instead, they represent functional CREs of gene expression.

## DISCUSSION

More than 90% of extant angiosperms retain evidence of one or more ancient genome‐wide duplications [68]. How TFs diversify in their DNA‐binding specificity and regulatory roles after gene duplications is of primary importance for understanding the functional complexity and environmental plasticity that emerged during plant evolution. The remarkable expansion of MYB family TFs accompanying plant terrestrialization has led to a large number of family members and a great intrafamily specificity divergence [69]. To understand how closely related members of AtMYBs recognize distinguished *cis‐*regulatory codes, this work comprehensively profiled the DNA‐binding specificity of subfamily VIII R2R3‐AtMYBs and found that homodimerization allows AtMYB2 to recognize a set of *cis*‐regulatory codes that are not bound by other AtMYBs (Figure 3A,C), which in turn enables AtMYB2 to bind (Figure 4F,H) and activate (Figure 5E and Figure S6F,H) specific target genes.

This finding offers an alternative solution to the “specificity paradox” faced by eukaryotic TFs. Prior studies have already suggested multiple solutions to resolve TFs with a similar monomeric motif, including (a) diversity in DNA shape preferences [18, 19, 51]; (b) combinatorial binding with different partners [70, 71, 72, 73, 74]; (c) multiple DNA‐binding specificities [75, 76]; (d) latent specificities induced by co‐factors [77, 78]; (e) DNA modifications [12, 13]; (f) binding to weak but specific sites in transcriptional hubs whereby the TF concentration is elevated [11]; (g) the configurations of TF homodimers [23, 25, 79]. In addition to these, here we show that homodimerization can also directly change the specificity of TFs and establish their unique DNA‐recognition selectivity. We therefore propose a dual‐role model through which homodimerization assists in discriminating between similar TFs (Figure 6D). This model also provides insights into gene editing—if homodimeric CREs with specificity changes are targeted, the editing will affect transcriptional regulation by only one of several paralogous TFs, thus improving the accuracy.

Subfamily VIII R2R3‐AtMYBs form a monophyletic clade that potentially originates from the same ancestral gene through duplications [30]. This study suggests that multiple mechanisms have cooperated to guide paralogous VIII AtMYBs to different *cis‐*regulatory codes. First, groups can be defined according to the identity of their primary motifs (Figure 1E). Next, within each group where the primary motifs are similar, the AtMYBs are further distinguished by a) more subtle monomeric specificity differences, such as the observed differences with the secondary/tertiary motifs (Figure 1E), and by b) the homodimeric binding preferences (Figure 6D), including the unique dimeric configurations (Figure 2A and Figure S3A) and the TF‐specific modifications of binding specificity upon homodimerization (Figure 3). Different from the compulsory dimeric TFs (e.g., bZIP, bHLH, MADS, and LFY), which bind dimeric sites with restricted orientations and short spacings, AtMYBs are capable of binding dimeric sites with fewer constraints. Such abundant homodimeric configurations of VIII AtMYBs also facilitated the discrimination between them (Figure 2A and Figure S3A).

The specificity profiles generated in this study also supplemented the previous DAP‐seq datasets [13] to illustrate the biochemical sequence preferences of AtMYBs, especially for their dimeric sequence preferences. From the data, we identified that the CCWAA‐box VIII AtMYBs have a distinct specificity and a high selectivity. Accordingly, these AtMYBs also share common structural features (Figure 1) and partially overlap regarding the physiological processes they regulate, such as stamen development [37, 80] and responses to the plant hormone ABA [34, 81]. Moreover, the generated data also inspire the rational design of CREs to drive tissue‐specific gene expression by VIII MYBs. The monomeric motif models generated for 16 VIII MYBs were not reported before. The motifs suggest that the previously identified consensus binding sequences were not optimal. For example, AtMYB2 was reported to bind A/TAACCA, C/TAACG/TG [34], TAACTG [80, 82, 83], and TAACGG [80]; in contrast, the generated motif models suggest that ACCTAAY has the highest affinity and should drive gene expression with the highest efficiency. In addition, the complex SELEX libraries also illustrate the dimeric preferences of VIII MYBs and provide a rich resource of dimeric motif models (Data S2). This facilitates the design of even stronger promoters and enhancers by offering the combinatorial rules of AtMYB CREs.

Altogether, through profiling the intact specificity landscape of AtMYBs, this work unraveled the intricacies and multiple sources of DNA‐binding specificities for closely related paralogous TFs beyond their seemingly analogous monomeric motifs and illustrated the versatile roles of homodimerization in establishing the unique identity of TFs and in enriching the complexity of the *cis‐*regulatory circuits.

## CONCLUSION

We investigated the specificity landscape of 40 R2R3‐MYBs of subfamily VIII using high‐throughput SELEX, curated 833 motif models and identified a cluster of “CCWAA‐box” AtMYBs that features a distinct specificity and a high selectivity. Moreover, with AtMYBs as an example, we demonstrated that homodimerization can evoke specificity changes—a yet unreported mechanism—to help distinguish between similar paralogous TFs. AtMYB2 has relied on this mechanism and developed a dimeric specificity that is distinct from other closely related “CCWAA‐box” AtMYBs and unique among all AtMYBs. The unique homodimeric *cis*‐regulatory codes of AtMYB2 are biochemically functional, conserved in evolution, and capable of activating transcription. Based on the facts, we proposed a dual‐role model through which homodimerization assists in discriminating between similar TFs.

## METHODS

### Cloning and protein expression

The full‐length CDS sequences of AtMYBs, the DBD sequences of AtMYB2/MA0073s0038 (*M. polymorpha*)/MN199019 (*S. moellendorffii*), and the progressive truncations of AtMYB2 (Table S1) were inserted into the pIX‐Halo with the In‐Fusion Kit (Vazyme; C112). Subsequently, the proteins with a Halo tag were expressed using the TnT® SP6 High‐Yield Wheat Germ Protein Expression System (Promega, L3261). For each AtMYB protein, 3 µg plasmids were used in a 50 µL reaction and incubated for 2 h at 25°C; the expressed proteins were then used for the subsequent experiments.

### High‐throughput SELEX and data pre‐processing

High‐throughput SELEX (HT‐SELEX) was performed as previously described [23]. The input SELEX library was designed to include a central random region of 101‐bp and flanking regions that were compatible with Illumina sequencing (TruSeq adapter sequences). Two microliters fusion proteins were incubated with 6 µL of SELEX library (30 ng/µL) and 30 µL of TCAPT buffer (140 mM KCl, 5 mM NaCl, 1 mM MgCl_2_, 3 µM ZnSO_4_, 100 µM EGTA, 10 mM Tris, pH 8, 0.1% Tween) at 25°C for 1 h. The DNA‐protein complexes were then enriched with Magne Halo‐Tag Beads (Promega, G7282), and the unbound DNA ligands were washed away with the HydroSpeed plate washer (Tecan, 30190101). The bound DNA ligands were then amplified by PCR with the amplification primers (Table S1) and used as the input library for the next cycle. After 3–5 cycles, the enriched DNA ligands and the input ligands were amplified with the PE primers (Table S1) and Illumina sequenced. Raw sequences from Illumina NovaSeq. 6000 were demultiplexed according to the i7 indices of each sample. Fastp [84] was used in pre‐processing, the low‐quality reads, and PCR duplicates were removed, the adaptors were trimmed, and then the paired‐end reads were merged and segmented into 40‐bp sequences for motif discovery.

### Data analyses of HT‐SELEX

Autoseed was used to *de novo* discover motifs from the pre‐processed SELEX reads with parameters “‐40N <Background sequence> <Signal sequence> 1 8 10 0.35–50 100” [85]. Autoseed constructs motifs based on the seed consensus and its neighboring kmers with Hamming distance ≤ 1, and therefore separates closely related models (Figure S1E). The motifs obtained from Autoseed were further filtered to remove duplicated motifs, single nucleotide runs, and motifs with total ICs <1. The spacing distance between two monomeric sites is defined by the distance between the two representative kmers [70]. For example, the spacing between two AtMYB2 monomers is defined by the distance intermediate of the two “RCCWAAY” sequences. The strength of TF signals in SELEX libraries was measured by E‐MI (enriched‐sequence‐based mutual information), which collectively captures all possible binding events of TFs [45]. The IC of the motifs was calculated by summing the IC values over all positions of a motif. Mean IC (per‐base IC) was calculated by dividing the IC of the motif by its length. The enrichment and frequency of all motifs in the SELEX library were evaluated by motif‐matching analyses. To compare the identified AtMYB motifs with previously reported plant motifs, all TF motifs in PlantPAN [6], PlantTFDB [48], and HOMER plants [49] were collected as the reference set. Classification and naming of MYB TFs were in accord with PlantTFDB [48]. Sequence alignment and phylogenetic analysis for MYB DBDs were performed respectively by mafft [86] and iqtree2 [87]. To calculate motif enrichments, motif matching analysis was performed using motifmatchr [88]. DNA shape analysis was performed using DNAshapeR [89]. ComplexHeatmap is used for heatmap visualization [90].

The dimeric preference of AtMYBs includes both the relative monomer orientation and spacing. The preference was analyzed by counting occurrences of ACCWAMY (a degenerated sequence representing the specificity of most AtMYBs) concatenated with different relative orientations and spacings. AlphaFold3 [91] was employed to build the structure models of AtMYB2‐DNA, using full‐length and DBD sequences of AtMYB2 and DNA sequences according to the consensus of each dimeric mode. Visualization of the structural models was achieved with PyMOL. The DNA contacts by AtMYB were visualized with DNAproDB [47]. To generate the in silico SELEX library, we first generated 10^7^ random sequences with a 101‐bp length. Then, the ER1 motif of AtMYB49 was matched to each random sequence with a sliding window, and the highest matching score among all windows was taken as the score of the sequence. Subsequently, the sequences with a score greater than 10 were collected as the in silico‐enriched SELEX library.

### Electrophoretic mobility shift assays (EMSA)

Double‐stranded DNA probes harboring the consensuses of Ma, ER1a, and ER8 of AtMYB2 were synthesized by annealing oligonucleotides and diluted to 10 ng/µL (DNA sequences in Table S2). Because the amount of protein expressed with the wheat germ system (Promega; L3261) is limited, here the AtMYB2 CDS sequence was inserted into pETG20A‐His and transformed into Rosetta 2 (DE3) pLysS strains [92]. The transformed *E. coli* was grown in LB medium (with auto‐induced ZYP5052 medium [23]) at 37°C for 4 h and 17°C for 14 h. The bacteria were then collected and lysed by lysis buffer (0.5 mg/mL lysozyme, 2 mg/mL DNase I, 1 mM PMSF), then incubated with His‐tag Ni Sepharose (Sangon, C600332) at 25°C for 2 h, and subsequently washed using Buffer A (50 mM Tris, 300 mM NaCl, 10 mM imidazole, pH 7.5) and Buffer B (Buffer A but with 50 mM imidazole) to remove nonspecifically bound proteins. Elution of the His‐tagged AtMYB2 protein was achieved using Buffer C (Buffer A with 500 mM imidazole). The purified AtMYB2 protein was concentrated using a 10 kD ultra‐centrifugal filter (Sigma; UFC8010). For EMSA, we prepared the binding buffer (10 mM Tris pH 7.5, 50 mM KCl) according to the commercial recipe (ThermoFisher, 20148) (Figure S1D). Subsequently, the AtMYB2 protein was diluted into 18, 24, 36, and 48 ng/µL gradient, and incubated with 10 ng DNA probes in a 20 µL reaction at 25°C for 1 h. The reaction mixture was loaded onto a 6% native PAGE and run in 0.5 × TBE at 4°C (100 min, 100 V). The gel was stained with gel blue (UElandy; S2019L) and imagined with a Bio‐Rad scanner (Bio‐Rad; 1708195EDU).

### DAP‐seq and data pre‐processing

DAP‐seq is performed as described [93, 94]. Briefly, genomic DNA was extracted from 4 weeks of *Arabidopsis thaliana* leaves and sonicated into 200‐bp fragments by the Covaris S2 sonicator, then purified using DNA clean beads (Vazyme; N411) at a 1.5:1 bead: DNA ratio. Adapters were then ligated to the genomic DNA fragments using the ligation toolkit (Vazyme; N203, N204) to generate the input library of DAP‐seq. Subsequently, 12 µL of Halo‐tagged AtMYB2/57/79 protein and 200 ng of the input library were incubated within 30 µL TCAPT buffer for 1 h. DNA‐protein complexes were pulled down with Magne Halo‐Tag Beads (Promega; G7282) and washed with the HydroSpeed plate washer (Tecan; 30190101). The DNA ligands bound to the AtMYB2/57/79 were amplified by PCR (using amplification primers in Table S1) and sequenced on an Illumina NovaSeq. 6000. Two independent biological replicates were included. Raw reads were demultiplexed, removed for low‐quality and adapter sequences, and then aligned using BWA v.0.7.17 [95] to the TAIR10 genome. The Araport11 files were used to obtain annotations of the genome.

### Data analyses of DAP‐seq

Peak calling was carried out using MACS3 v.3.0.0a7 [96] with parameters “‐f BAMPE ‐‐d‐min 5 ‐‐min‐length 15 ‐‐call‐summits ‐‐keep‐dup all ‐‐cutoff‐analysis.” The motif hits in DAP‐seq libraries were identified by motifmatchr [88] with the *p*‐value set to 1e‐5. To calculate enrichments, the motif hits in shuffled DAP‐seq peaks were used as the background. A PR analysis was conducted by utilizing motifmatchr to score the 200‐bp genomic fragments. The fragments overlapping with the top 3000 DAP‐seq peaks were used as the positive set, while the remaining fragments served as the negative set. The highest motif‐matching score across the 200‐bp region is taken as the score of a segment. By varying the threshold of the binary classifier, the PR curves were generated, and the AUPRC values were calculated. Analyses for the dimeric configurations in DAP‐seq libraries were performed the same as described for the SELEX libraries. To obtain less biased motifs, and to enable comparison with SELEX, here we also used Autoseed (with parameters “‐40N <Background sequence> <Signal sequence> 1 8 10 0.35 ‐ 50 100”) to discover motifs for DAP‐seq libraries.

### Overexpression of AtMYBs in *Arabidopsis* protoplasts

The full‐length CDS sequence of AtMYB2/57/79 was inserted into the pHBT‐GFP‐NOS, which was then transferred into *Arabidopsis* (Col‐0 ecotype) protoplasts to overexpress the AtMYBs protein. Protoplasts were isolated from leaves of 4‐week‐old *Arabidopsis* and transfected [97]. The isolated protoplasts were resuspended in the MMG solution (0.4 M mannitol, 15 mM MgCl_2_, 4 mM MES, pH 5.7) to a concentration of 2 × 10^5^ cells/mL. Transfection was performed by combining 1 mL protoplasts with 100 µL plasmid (1 µg/µL) and 1.1 mL PEG solution (40% PEG4000, 0.2 M mannitol, 0.1 M CaCl_2_). After 8 min, transfection was stopped by adding 4.4 mL of W5 solution (154 mM NaCl, 125 mM CaCl_2_, 5 mM KCl, and 2 mM MES, pH 5.7). The transfected protoplasts were incubated for 14 h at room temperature to allow protein expression, followed by centrifuging to collect 5 × 10^6^ cells as the AtMYB2 overexpression sample for RNA‐seq and MNase‐seq.

### RNA‐seq and data analyses

5 × 10^6^ cells of AtMYB‐overexpressed protoplasts were collected for RNA‐seq. RNA isolation, library construction, and sequencing were performed by the Novogene Company. The obtained RNA‐seq raw reads were first removed for adapter sequences, and low‐quality reads by Trimmomatic v.0.39 [98]. The filtering parameters are “ILLUMINACLIP:TruSeq. 3‐PE‐2.fa:2:30:7:1 LEADING:3 TRAILING:3 SLIDINGWINDOW:4:13 MINLEN:50.” Then, the clean reads were mapped to the TAIR10 genome using Hisat2 v.2.2.1 [99]. The mapped reads from each RNA‐seq library were next assembled into transcriptome by Stringtie v.1.3.4 with the parameter “‐G Araport11.gtf” and removed for redundancy by CD‐HIT v.4.7. Then the genomic sequences of the assembled transcriptome were extracted into a fasta file, and indexed with salmon v.0.13.1 [100]. With the index, the expression of genes was calculated using the fastq files generated by Trimmomatic, and normalized to TPM (transcripts per million reads) with salmon. Subsequently, genes with false discovery rate (FDR) < 0.05 and |log_2_fold change| > 2 were defined as differentially expressed genes using DESeq. 2. GO analyses were then carried out with GO information from org.At.tair.db.

### Conservation and TSS‐enrichment analyses of AtMYB2 CREs

To visualize the distribution of AtMYB2 CREs near the TSSs of the *A. thaliana* genome, MYB2 motifs were used to match the genomic sequences using motifmatchr [88] with the *p*‐value set to 1e‐4. To eliminate potential biases from the nucleotide composition of the motifs, the distributions of reversed AtMYB2 CREs (matches of the reversed motifs) were subtracted from the distribution of AtMYB2 CREs. The resulting distribution profiles were further smoothed with a 200‐bp window. For conservation analysis, the genome‐wide PhastCons scores of *Arabidopsis* calculated across 63 representative plants were downloaded from the PlantRegMap [67] database. AtMYB2 CREs (motif hits) in the promoters (0 to −1 kb from TSSs) were included in the conservation analysis, and the average conservation scores around the aligned AtMYB2 CREs were calculated for each position and visualized.

### Dual‐luciferase reporter assay

The CDS sequences of AtMYBs were inserted into the pHBT‐GFP‐NOS vector for expression of AtMYB2/57/79. The reporter vector pGreenII 0800‐LUC was constructed [101] to include four repeats of the consensus of the AtMYB2 binding motifs from SELEX, followed by a mini35S promoter (DNA sequences in Table S2). The plasmids were extracted with the GoldHi EndoFree Plasmid Maxi Kit (CWBIO, CW2104M) and transfected into protoplasts as described above. The protoplasts were placed in the dark for 12 h at room temperature. Next, the luminescence strengths were measured using a reporter assay kit (Yeasen; 11402ES60) and a microplate reader (Tecan, Spark). The ratio of Fluc to Rluc was calculated to represent promoter activity.

### MNase‐seq and data analyses

MNase‐seq follows the described procedure [102], 5 × 10^6^ cells of *Arabidopsis* protoplasts were collected for MNase‐seq. 1 ml of chilled RIPA buffer (10 mM Tris–HCl, pH 8.0, 140 mM NaCl, 1 mM EDTA, 1% Triton X‐100, 0.1% SDS, 0.1% sodium deoxycholate, 0.25 M sucrose, 0.1% beta‐ME, 1 mM PMSF) was added. After a thorough vortex, the mixture is centrifuged at 800 *g* for 2 min to remove the supernatant. The washing process is repeated 3–5 times. The final pellet containing nuclei was resuspended by 200 µL MNase buffer (0.3 M sucrose, 20 mM Tris‐HCl, pH 7.5, 3 mM CaCl_2_). The Micrococcal Nuclease (New England Biolabs, M0247S) was diluted 128 times with the MNase buffer, and 3 µL of the diluted MNase was added into 200 µL resuspension of the nuclei, incubated at 37°C and 1500 rpm for 8 min on a mixer (Miulab; MTH‐100). The digestion was terminated by adjusting the EDTA concentration to 20 mM. The digested DNA fragments were recovered using DNA purification beads (Vazyme, N411), and subjected to Illumina library preparation. MNase‐seq raw reads were removed for low‐quality sequences and adapter sequences using Trim Galore with parameters “‐q 30 ‐‐paired ‐‐stringency 5 ‐‐fastqc ‐‐gzip”. The BWA aligner [95] was used to align the reads to the TAIR10 genome with the quality threshold set to 20. The alignments are then converted to bam format, sorted and removed for duplicates, indexed, and visualized.

### Statistical analyses

The statistical analyses were performed in R. The function wilcox.test was used to apply the Wilcoxon signed‐rank test. Fisher's exact test was performed with the fisher.test function. *T*‐tests were performed with the t.test function.

## AUTHOR CONTRIBUTIONS

**Tian Li**: Writing—review and editing; data curation; investigation; validation; formal analysis; visualization. **Hao Chen**: Writing—review and editing; data curation; investigation; validation; formal analysis; visualization. **Nana Ma**: Data curation. **Dingkun Jiang**: Data curation. **Jiacheng Wu**: Data curation. **Xinfeng Zhang**: Data curation. **Hao Li**: Data curation. **Jiaqing Su**: Data curation. **Piaojuan Chen**: Data curation. **Qing Liu**: Data curation. **Yuefeng Guan**: Project administration. **Xiaoyue Zhu**: Project administration. **Juncheng Lin**: Project administration. **Jilin Zhang**: Project administration. **Qin Wang**: Project administration. **Honghong Guo**: Writing—review and editing; formal analysis; visualization; funding acquisition. **Fangjie Zhu**: Writing—review and editing; investigation; validation; formal analysis; funding acquisition.

## CONFLICT OF INTEREST STATEMENT

The authors declare no conflicts of interest.

## ETHICS STATEMENT

No animals or humans were involved in this study.

## Supporting information


**Figure S1:** Closely related R2R3‐AtMYBs bind highly similar sequences.
**Figure S2:** HT‐SELEX enriches AtMYBs binding signals.
**Figure S3:** Modified specificities of closely spaced AtMYBs homodimers.
**Figure S4:** Enrichment of AtMYB2 binding modes in MYBs of other plants.
**Figure S5:** AtMYB2 recognizes unique targets with modified specificity.
**Figure S6:** The homodimeric binding modes of AtMYB2 activate transcription.
**Figure S7:** Transcriptional regulation by AtMYB2.
**Data S1:** *De novo* motifs from published DAP‐seq libraries of VIII AtMYBs.
**Data S2:** Monomeric and homodimeric motifs of VIII R2R3‐AtMYBs.

**Table S1:** Sequence information for DNA ligands.
**Table S2:** Sequence information for dual‐luciferase reporter assay and EMSA.
**Table S3:** Orthologs of AtMYB2.
**Table S4:** MYB2 motif models from SELEX library.
**Table S5:** MYB57 motif models from SELEX library.
**Table S6:** MYB79 motif models from SELEX library.
**Table S7:** MYB90 motif models from SELEX library.
**Table S8:** MYB19 motif models from SELEX library.
**Table S9:** MYB46 motif models from SELEX library.
**Table S10:** MYB50 motif models from SELEX library.
**Table S11:** MYB61 motif models from SELEX library.
**Table S12:** MYB67 motif models from SELEX library.
**Table S13:** MYB83 motif models from SELEX library.
**Table S14:** MYB80 motif models from SELEX library.
**Table S15:** MYB49 motif models from SELEX library.
**Table S16:** MYB107 motif models from SELEX library.
**Table S17:** MYB63 motif models from SELEX library.
**Table S18:** MYB74 motif models from SELEX library.
**Table S19:** MYB13 motif models from SELEX library.
**Table S20:** MYB58 motif models from SELEX library.
**Table S21:** MYB10 motif models from SELEX library.
**Table S22:** MYB30 motif models from SELEX library.
**Table S23:** MYB30 motif models from SELEX library.
**Table S24:** MYB121 motif models from SELEX library.
**Table S25:** MYB27 motif models from SELEX library.
**Table S26:** MYB62 motif models from SELEX library.
**Table S27:** MYB108 motif models from SELEX library.
**Table S28:** MYB21 motif models from SELEX library.
**Table S29:** MYB71 motif models from SELEX library.
**Table S30:** MYB66 motif models from SELEX library.
**Table S31:** MYB14 motif models from SELEX library.
**Table S32:** MYB72 motif models from SELEX library.
**Table S33:** MYB114 motif models from SELEX library.
**Table S34:** MYB41 motif models from SELEX library.
**Table S35:** MYB34 motif models from SELEX library.
**Table S36:** MYB85 motif models from SELEX library.
**Table S37:** MYB51 motif models from SELEX library.
**Table S38:** MYB99 motif models from SELEX library.
**Table S39:** MYB15 motif models from SELEX library.
**Table S40:** MYB55 motif models from SELEX library.
**Table S41:** MYB86 motif models from SELEX library.

## Data Availability

The data that support the findings of this study are openly available in the China National Genomics Data Center at https://ngdc.cncb.ac.cn/gsa/browse/CRA014337. All sequencing data have been deposited to China National Genomics Data Center under accession PRJCA022556, with raw sequence data under GSA: CRA014337 (https://ngdc.cncb.ac.cn/gsa/browse/CRA014337). Data link for UCSC Genome Browser session: https://genome.ucsc.edu/s/CH_1997/MYB2_project. The scripts have been deposited on GitHub (https://github.com/Daven-1997/2025_R2R3_MYBs_code). Supplementary materials (figures, tables, graphical abstract, slides, videos, Chinese translated version, and updated materials) may be found in the online DOI or iMeta Science http://www.imeta.science/.
