# Supplementary material for: Specificity landscapes of 40 R2R3‐MYBs reveal how paralogs target different cis‐elements by homodimeric binding
Source: Imeta. 2025 Mar 5;4(2):e70009. doi: 10.1002/imt2.70009 (PMC11995187; doi:10.1002/imt2.70009)
Supplement: Supplementary file 1 — Figure S1: Closely related R2R3‐AtMYBs bind highly similar sequences. Figure S2: HT‐SELEX enriches AtMYBs binding signals. Figure S3: Modified specificities of closely spaced AtMYBs homodimers. Figure S4: Enrichment of AtMYB2 binding modes in MYBs of other plants. Figure S5: AtMYB2 recognizes unique targets with modified specificity. Figure S6: The homodimeric binding modes of AtMYB2 activate transcription. Figure S7: Transcriptional regulation by AtMYB2. Data S1: De novo motifs from published DAP‐seq libraries of VIII AtMYBs. Data S2: Monomeric and homodimeric motifs of VIII R2R3‐AtMYBs. [file IMT2-4-e70009-s002.docx]

**Supporting information to**

**Specificity landscapes of 40 R2R3-MYBs reveal how paralogs target different *cis*-elements by homodimeric binding**

**Running title**: Homodimerization helps differentiate the *cis*-elements of paralogous AtMYBs

Tian Li^1#^, Hao Chen^1#^, Nana Ma^1,2#^, Dingkun Jiang^1,2^, Jiacheng Wu^1^, Xinfeng Zhang^1^, Hao Li^1,2^, Jiaqing Su^3^, Piaojuan Chen^1^, Qing Liu^4^, Yuefeng Guan^3^, Xiaoyue Zhu^1^, Juncheng Lin^1^, Jilin Zhang^5,6,7^, Qin Wang^1*^, Honghong Guo^1,2*^, Fangjie Zhu^1*^

^1^Haixia Institute of Science and Technology, National Engineering Research Center of JUNCAO, College of JUNCAO Science and Ecology, Fujian Provincial Key Laboratory of Haixia Applied Plant Systems Biology, Fujian Agriculture and Forestry University, Fuzhou, 350002, China

^2^College of Life Science, Fujian Agriculture and Forestry University, Fuzhou, 35002, China

^3^College of Resources and Environment, Fujian Agriculture and Forestry University, Fuzhou, 350002, China

^4^State Key Laboratory for Conservation and Utilization of Subtropical Agro-Bioresources, South China Agricultural University, Guangzhou, 510640, China

^5^Department of Biomedical Sciences, City University of Hong Kong, Hong Kong, 999077, China

^6^Tung Biomedical Sciences Centre, City University of Hong Kong, Hong Kong, 999077, China

^7^Department of Precision Diagnostic and Therapeutic Technology, The City University of Hong Kong Shenzhen Futian Research Institute, Shenzhen, 518057, China

^#^These authors contributed equally: Tian Li, Hao Chen, Nana Ma

^*^Correspondence: [fjzhu@fafu.edu.cn](mailto:fjzhu@fafu.edu.cn) (Fangjie Zhu), [guohh@fafu.edu.cn](mailto:guohh@fafu.edu.cn%20) (Honghong Guo), [qinwangCRY@163.com](mailto:qinwangCRY@163.com) (Qin Wang).

**
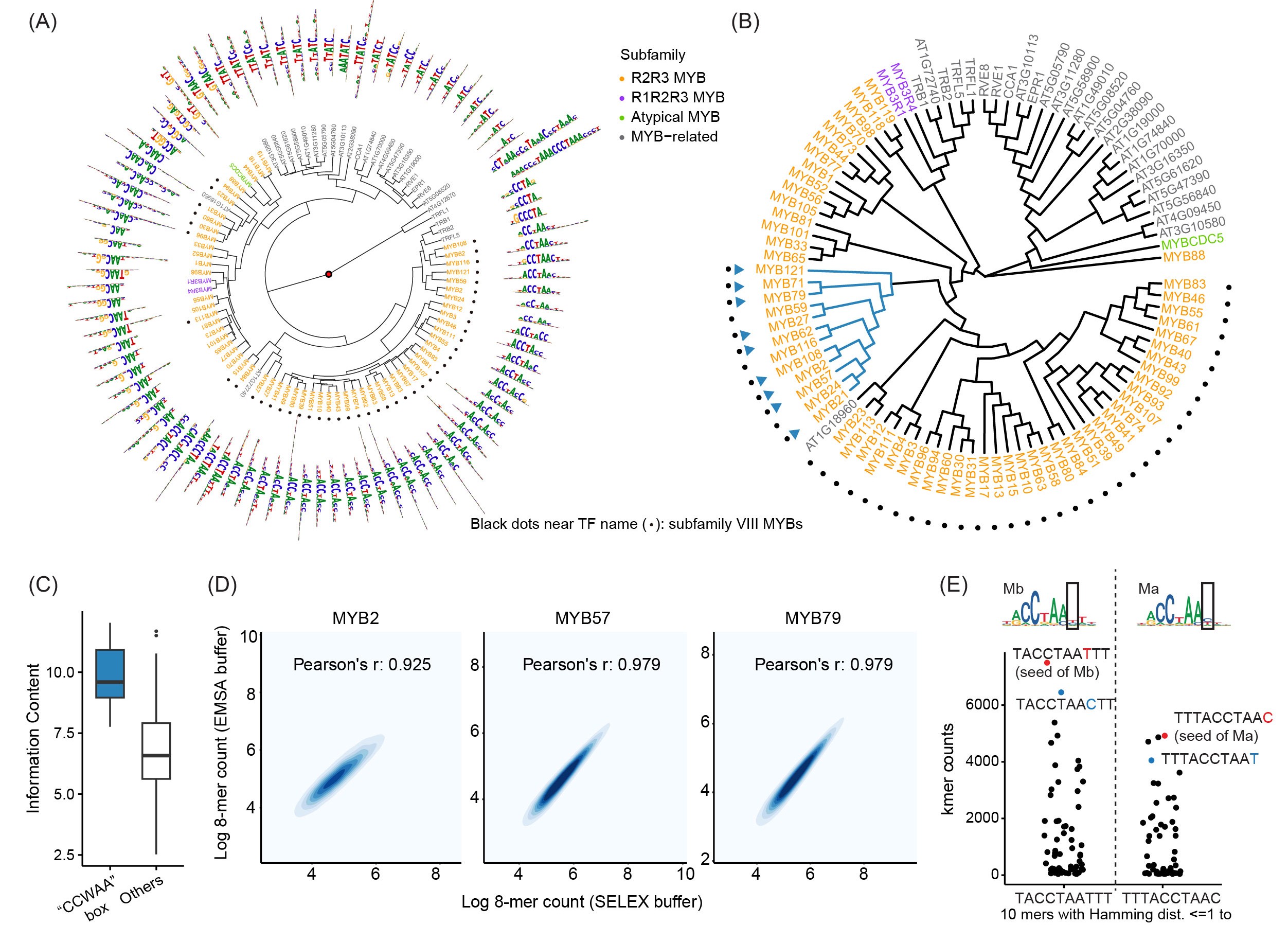
**

**Figure S1 Closely related R2R3-AtMYBs bind highly similar sequences.** (A–B) Phylogenetic analysis of R2R3-AtMYBs motifs (A) and DBD sequences (B). Note that R2R3-AtMYBs with similar DBDs tend to bind similar DNA sequences. Blue color highlights the CCWAA-box AtMYBs, they share similar DBD sequences (B) and bind to similar monomeric motifs in SELEX (Figure 1E). The motifs of R2R3-AtMYBs (A) were from public databases [1–3]. (C) CCWAA-box AtMYBs are more selective than other AtMYBs. The information contents of CCWAA-box and other AtMYBs were shown. (D) Highly similar DNA binding specificity of AtMYBs in EMSA and SELEX buffer. Density plots showing log 8-mer counts in SELEX libraries enriched with EMSA (50 mM KCl) and SELEX (140 mM KCl, TCAPT buffer) buffers. (E) Separation of closely related motif models. For the two similar motifs (Ma and Mb) of AtMYB2, counts of their seeds and the neighboring 10-mers (hamming distance to seed <= 1) used in motif construction are shown. The minor differences between Ma and Mb of AtMYB2 reveal the relative enrichment of local 10-mers. For example, the order of T and C for the “Y” position of ACCTAAY is reversed between Ma and Mb. This is explained by the kmer counts that TTTACCTAAC > TTTACCTAAT (Ma), and that TACCTAATTT > TACCTAACTT (Mb).

**
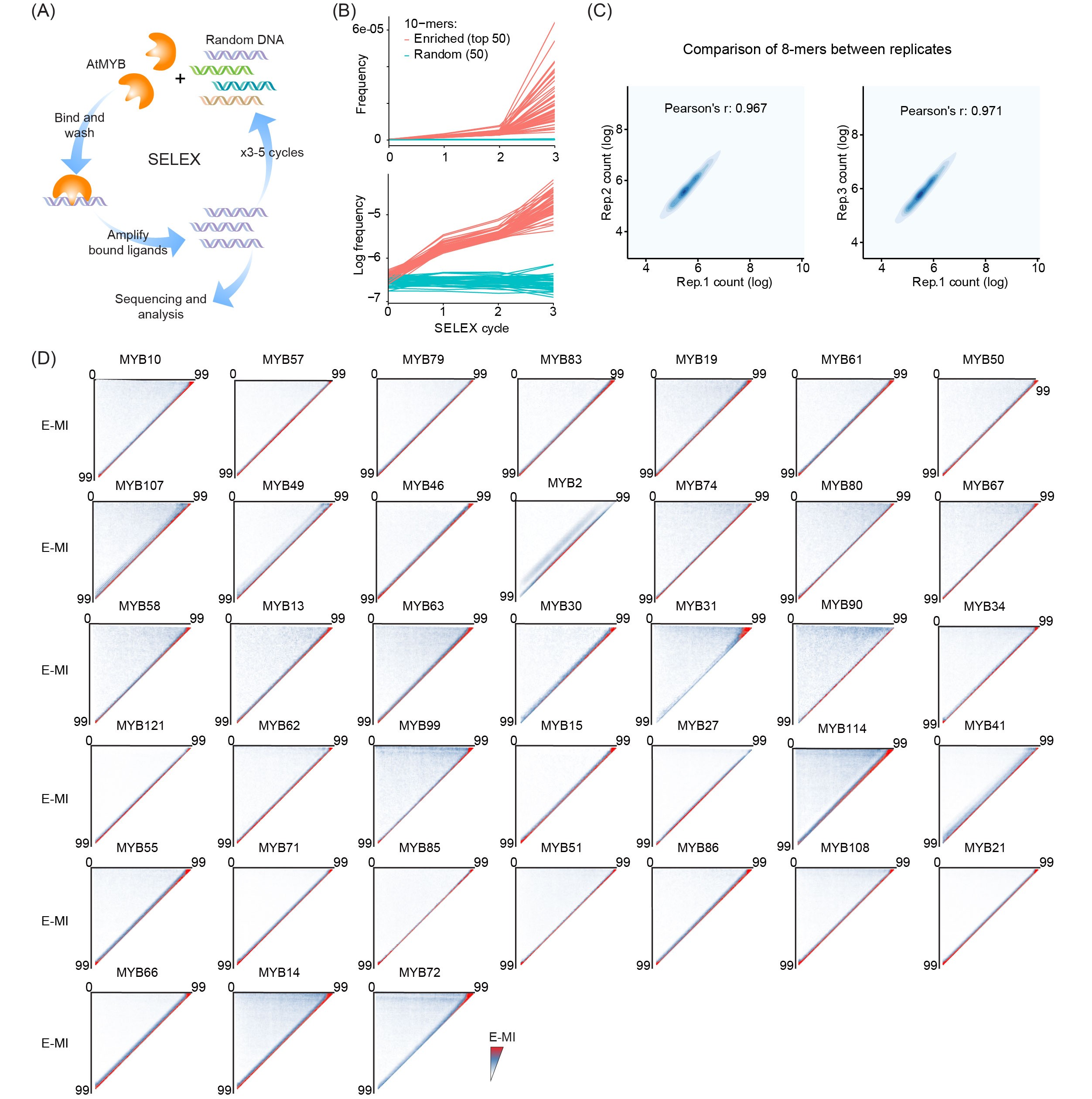
**

**Figure S2 HT-SELEX enriches AtMYBs binding signals.** **(**A) Schematic of the HT-SELEX. The double-stranded DNA library with a 101-bp random region was incubated with each AtMYB protein. The unbound DNA ligands were removed by washing, the bound DNA ligands were amplified by PCR and used for the next cycle. After 3–5 cycles, the enriched DNA ligands were sequenced. (B) The bound DNA ligands were exponentially enriched. The frequencies of subsequences (10-mers) in each cycle of AtMYB2 SELEX were plotted. In addition to the bound sequences (top 50), 50 randomly selected sequences were also visualized as the control. (C) The replicates in SELEX agree with each other. Log 8-mer counts (density visualized) between pairs of AtMYB2 SELEX replicates. (D) Binding signals of AtMYBs in the SELEX libraries. E-MI signals near the hypotenuse of the triangle become stronger than elsewhere, suggesting that TF signals have been enriched in each SELEX library. In total successful libraries were obtained for 38 AtMYBs in this study.

**
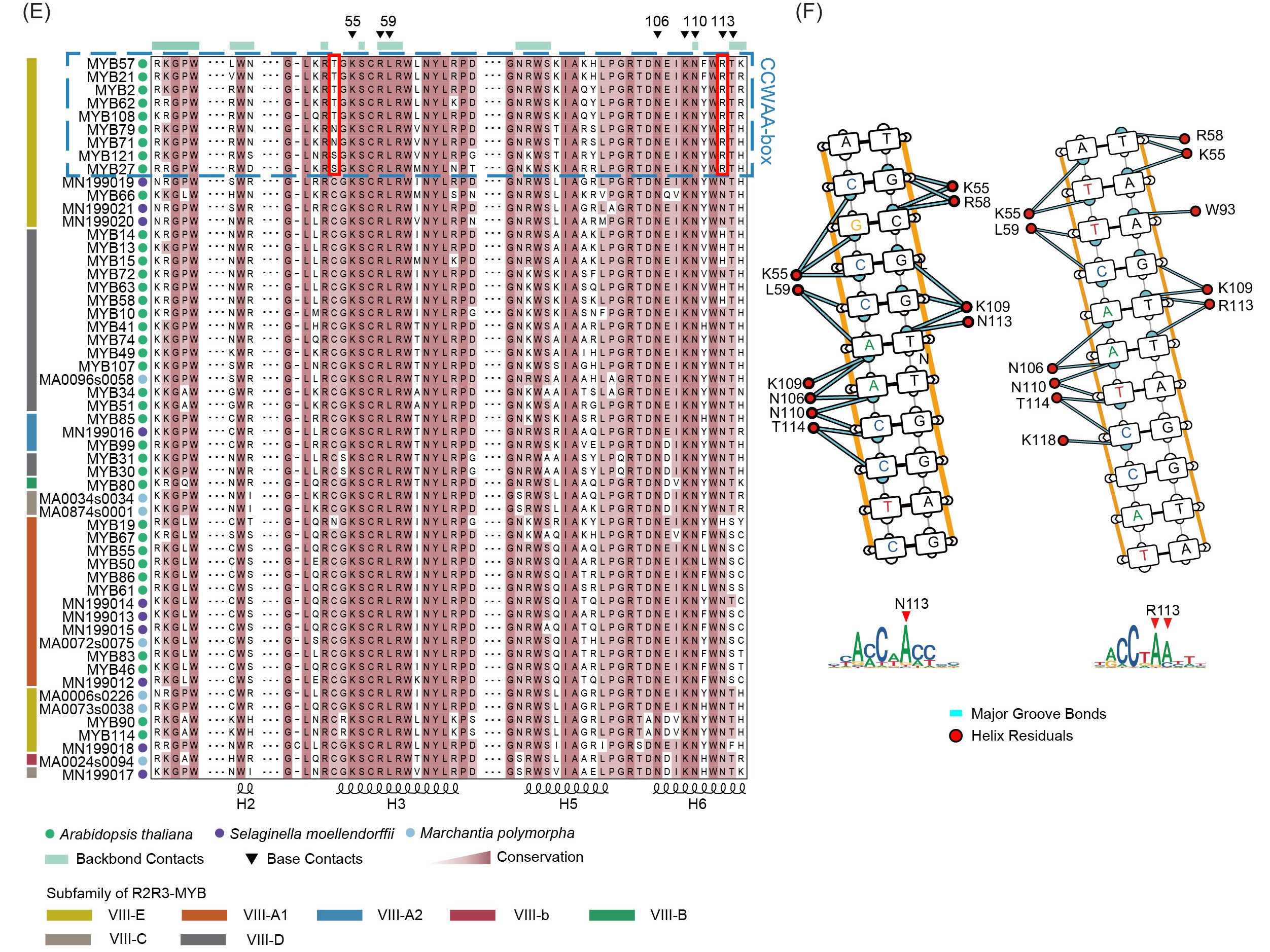
**

**Figure S2 HT-SELEX enriches AtMYBs binding signals.** (E) Sequence alignment of the DBDs of R2R3-MYBs. The VIII AtMYBs studied in this work are aligned with all VIII MYBs in *M. polymorpha* and *S. moellendorffii*. DNA-contacting residuals are annotated based on the structure of AtMYB66 (PDB: 6KKS [4], also included in alignment). Red boxes highlight the mutated residuals that likely account for the distinct specificity of the CCWAA-box AtMYBs (blue box) because these residuals in CCWAA-box AtMYBs are different compared to most other VIII AtMYBs and VIII MYBs in ancestor species. (F) AtMYB2 and AtMYB66 both contact DNA bases with position 113. The crystal structure of AtMYB66 and AlphaFold structure of AtMYB2 were visualized for contacts. Note that R113 (AtMYB2) contacts 2 “A” bases (red triangle), while N113 (AtMYB66) only contacts 1 “A” base.

**
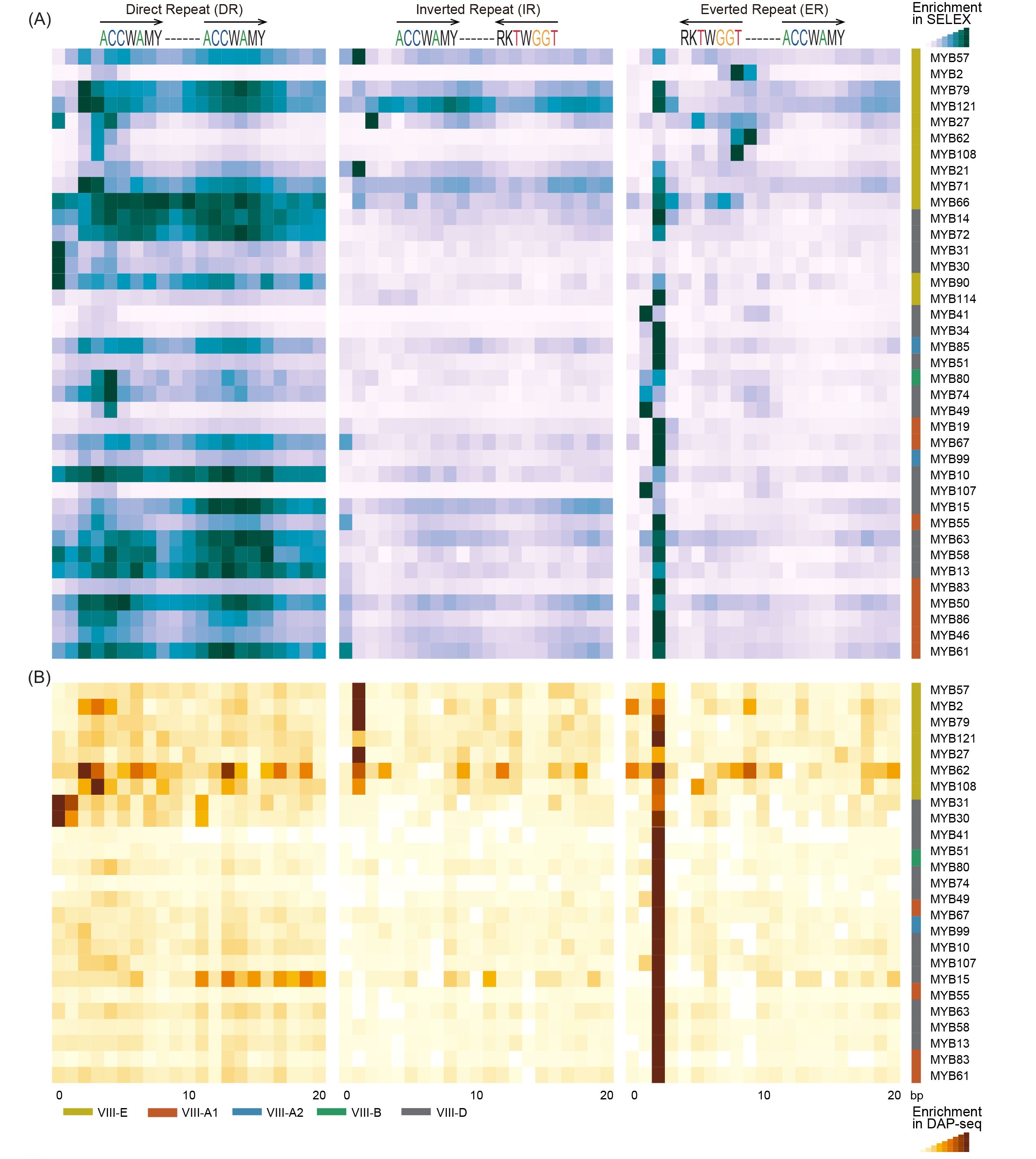
**

**Figure S3 Modified specificities of closely spaced AtMYBs homodimers.** (A) AtMYBs with similar monomeric motifs have different homodimeric preferences. Enrichments of dimeric AtMYB CREs with different spacings and three relative orientations in SELEX libraries. Two “ACCWAMY” strings are concatenated with the annotated spacing (x-axis) and assessed for enrichment. (B) Homodimeric preferences of genomic AtMYB CREs. Enrichments of dimeric AtMYB CREs with different spacings and three relative orientations in DAP-seq peaks.

**
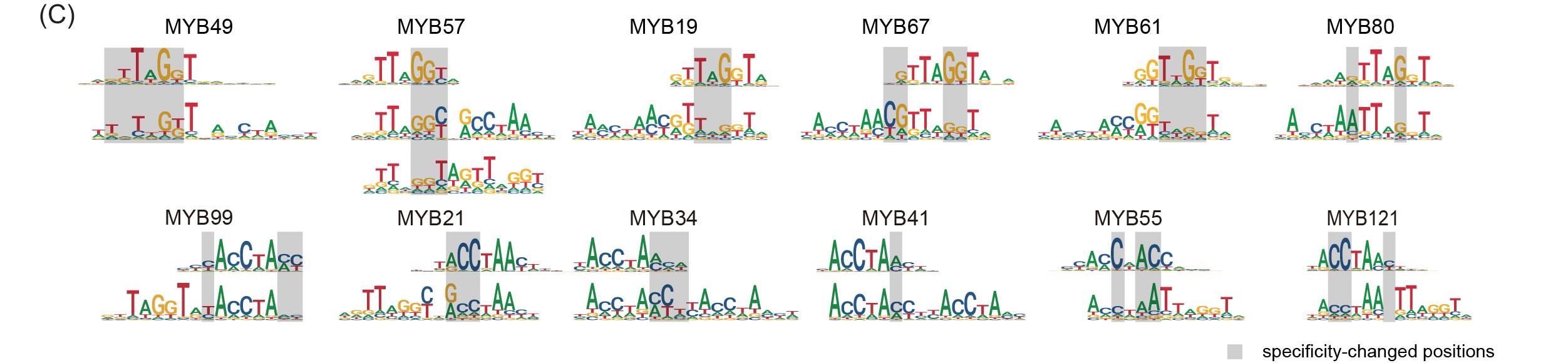
**

**
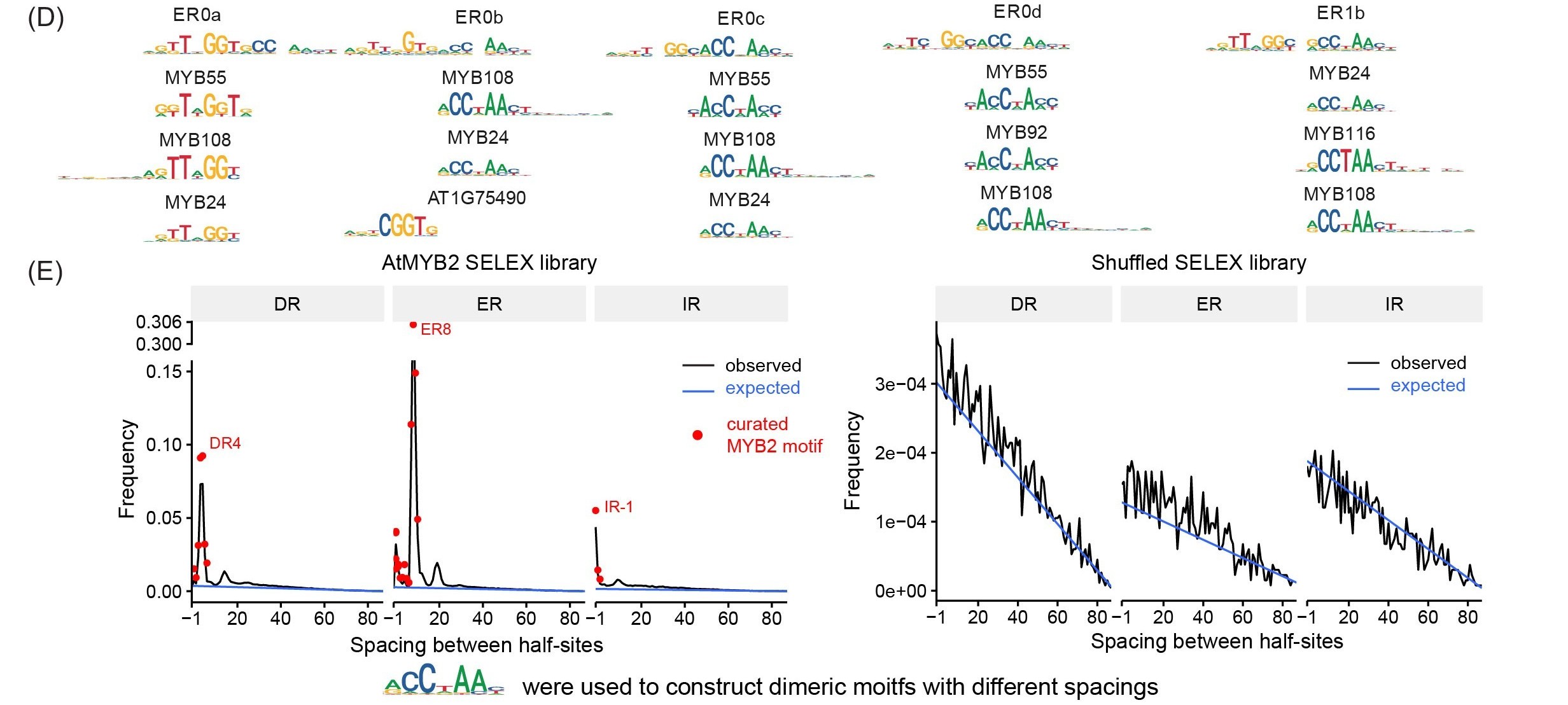
**

**Figure S3 Modified specificities of closely spaced AtMYBs homodimers.** (C) Closely spaced AtMYB homodimers alter its DNA-binding specificity. Half-sites in the closely spaced dimeric motifs of AtMYBs deviate from their monomeric motifs. (D) ER0 and ER1 modes of AtMYB2 are dissimilar to reported plant motifs. The ER motifs were compared to all reported plant motifs [1–3], and the three most similar known motifs are shown. (E) Cooperativity of the dimeric binding of AtMYB2. To examine whether the dimeric modes with unchanged half-sites are cooperative, the core of Ma (indicated at the bottom) is used to construct dimeric motifs with different spacings/orientations, and their frequencies are visualized (black lines, motif match *p* = 1e-6). To test the cooperativity, we use a linear fit to estimate the expected frequency of non-cooperative bindings (blue lines). The linear fit relies on dimeric frequencies spaced by 67–87 bp because, with such a distance, the binding of two monomers is less likely to cooperate. The fit describes well the non-cooperative distribution of two monomeric motifs (right panel) and suggests that closely-spaced homodimers are in general cooperative (left panel). The curated AtMYB2 motifs (red points) also feature higher frequencies than non-cooperative bindings (blue lines).

**
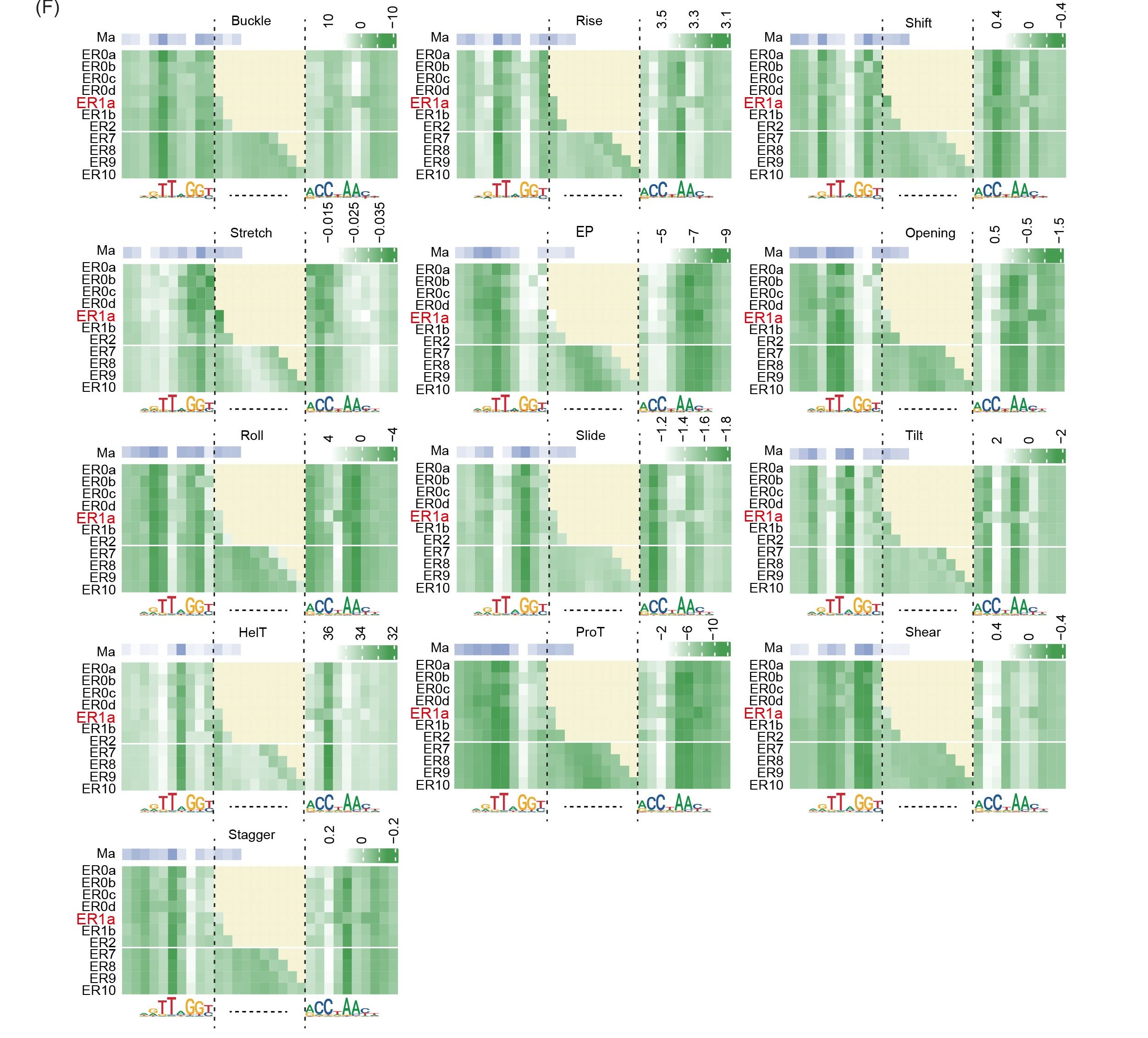
**

**
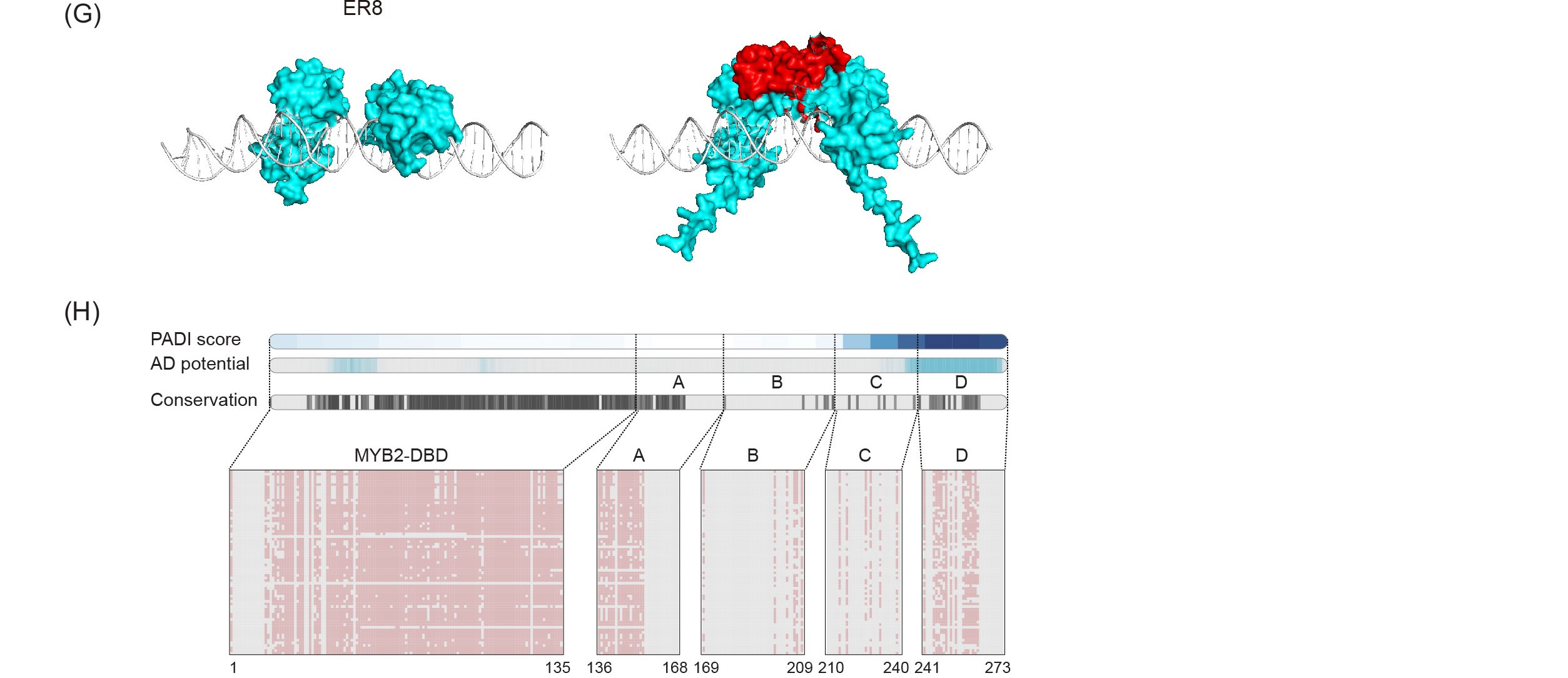
**

**Figure S3 Modified specificities of closely spaced AtMYBs homodimers.** (F) Distinct DNA-shape preference of AtMYB2 ER1a. Average DNA-shape parameters are illustrated for each position of the sequences harboring respective motifs of AtMYB2. Positions are aligned with additional gaps (the light yellow zone between the vertical dashes) to separate the two half-sites of ER0–2. Note that the profile is similar for half-sites of all motifs except for ER1a. (G) The non-DBD region of AtMYB2 forms contacts when bound to ER8. AlphaFold structures show that when bound to ER8, only the full-length AtMYB2 (right panel) but not its DBD (left panel) can form protein-protein contacts. The non-DBD region is colored red. Loop regions are hidden. (H) Sequence conservation of AtMYB2 orthologs. AA sequences of AtMYB2 and its 70 orthologs (identified using OrthoFinder [5]) from 66 eudicots (from Ensembl Plants; Table S3) were aligned to examine sequence conservation. Amino acids conserved in more than half of the orthologs are colored pink. The degree of conservation is also summarized across AtMYB2. The activation domain (AD) potential (from ADpred [6]) and plant activation domain identification (PADI) score [7] were shown across AtMYB2 to indicate the location of the activation domain. Note that segments A and D in the non-DBD region are more conserved than segments B and C, this is in accord with the more drastic changes with dimeric preference when truncating A and D (Figure 2F).


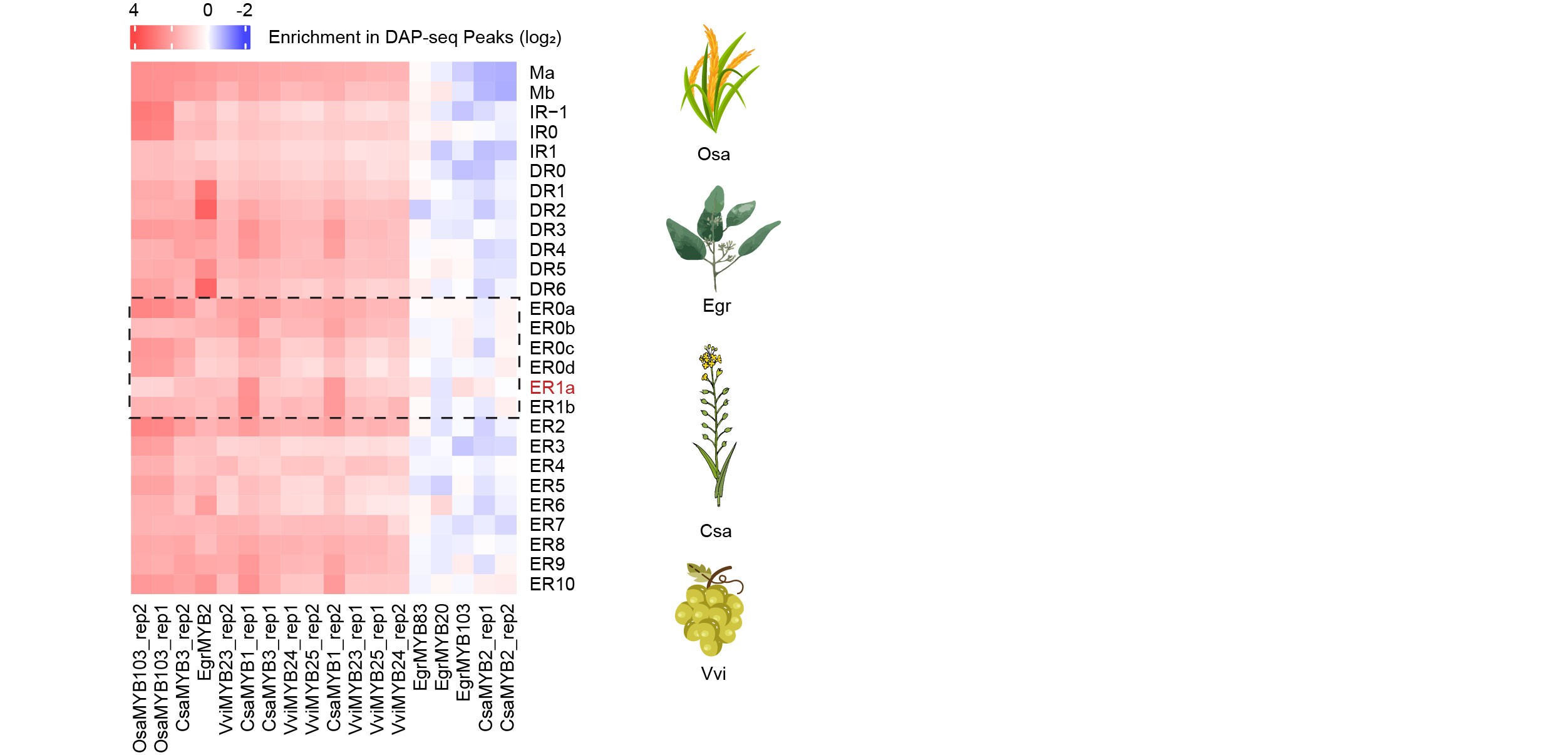


**Figure S4 Enrichment of AtMYB2 binding modes in MYBs of other plants.** Enrichments of AtMYB2 motifs are surveyed for DAP-seq peaks of MYB TFs of other plants, including *Eucalyptus grandis* (Egr), *Oryza sativa* (Osa), *Vitis vinifera* (Vvi) and *Camelina sativa* (Csa).


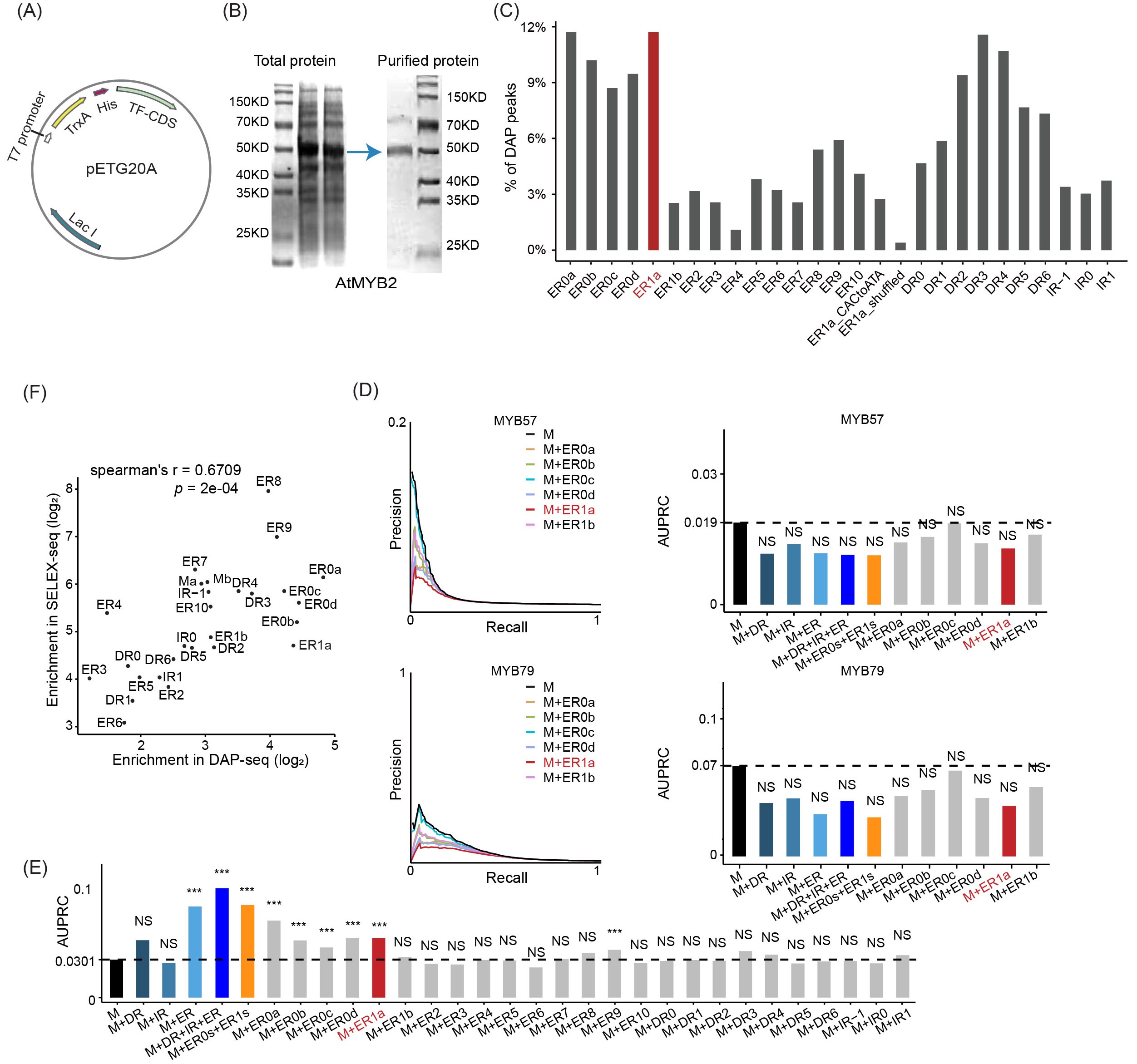


**Figure S5 AtMYB2 recognizes unique targets with modified specificity.** (A) The construction of expression vector. The full-length fragment of AtMYB2 CDS was inserted into pETG20A. (B) Protein purification. SDS-PAGE shows that high-purity AtMYB2 proteins were obtained after His-tag-based purification. (C) Fraction of DAP peaks containing dimeric AtMYB2 motifs. ER1a (red) is among the most enriched dimeric modes in the top 3000 peaks of DAP-seq. (D) ER0s and ER1s cannot predict genomic CREs of AtMYB57/79. Precision-recall curves (left) and the area under the curve (AUPRC, right) for motif-based prediction of AtMYB57/79 DAP-seq peaks. Note that when combined with the monomeric motifs (M), the ER1s and ER0s of AtMYB2 have decreased the predictive power. This is in contrast with AtMYB2, whereby ER0s and ER1s increased the predictive power (Figure 4C–E and Figure S5E). Wilcoxon signed-rank test (for paired precision values at each recall level) is performed to evaluate if adding the dimeric modes to M has significantly increased the predictive power. (E) ER0s and ER1s predict genomic CREs of AtMYB2. The area under the curve (AUPRC) values for motif-based prediction of AtMYB2 DAP-seq peaks. Note that when combined with the monomeric motifs (M), ER1s and ER0s have significantly increased the predictive power. Statistical tests were performed as in (D). (F) ER0s and ER1s are more enriched in DAP-seq than in SELEX. Enrichments of AtMYB2 motifs in SELEX and DAP-seq correlate with each other, but with minor discrepancies that likely reflect the preferred sequence usage by the genomic CREs.


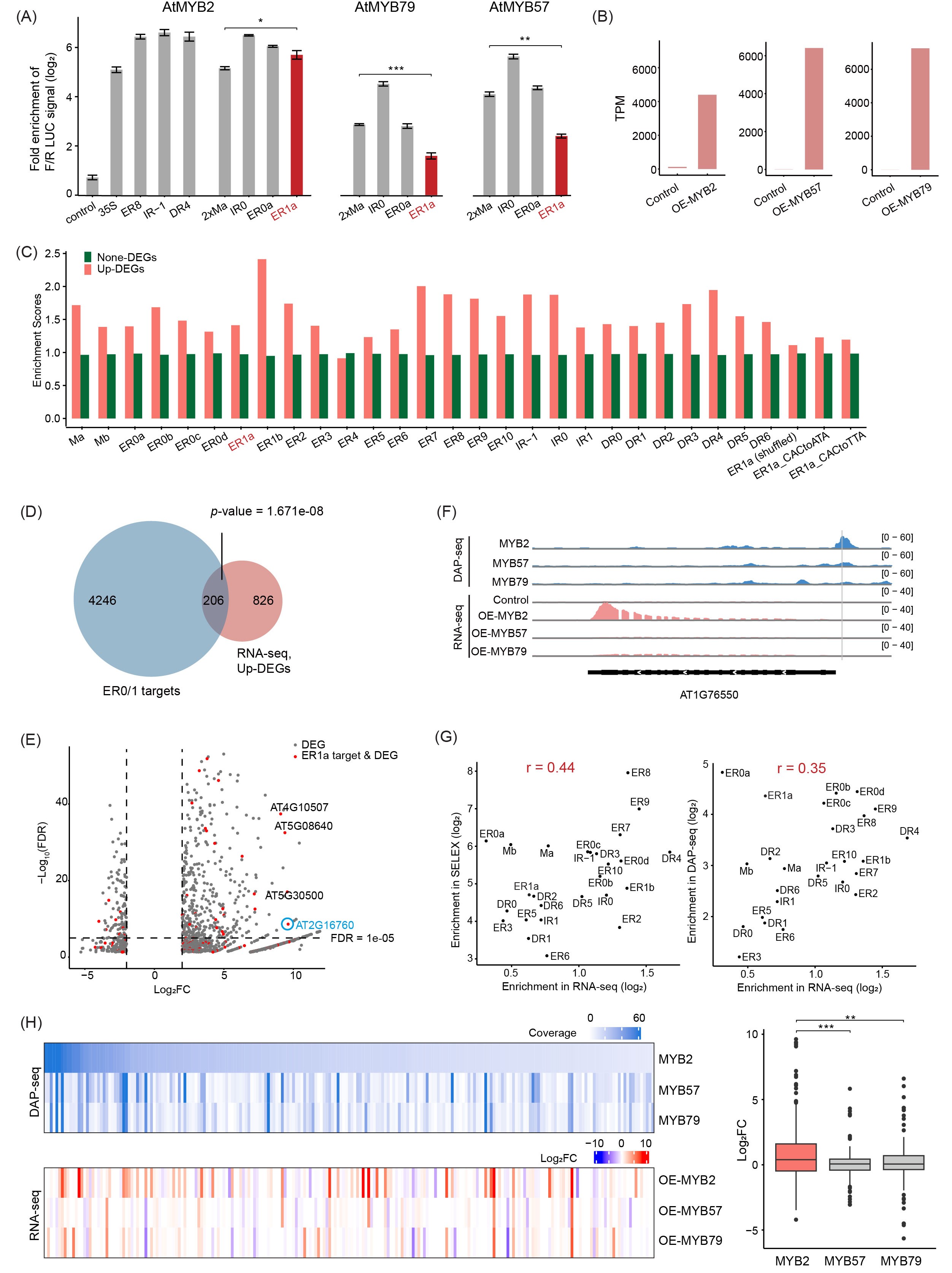


**Figure S6 The homodimeric binding modes of AtMYB2 activate transcription.** (A) ER0/1s are transcriptionally active only for AtMYB2. Dual-luciferase reporter assays with 4 × repeats of AtMYB2 motifs. Note that the high-affinity dimeric modes identified by SELEX (IR0/-1, DR4, ER8, ER0/1s) are more active than 2 copies of the monomeric sites (2 × Ma) and that ER0/1s are much less active for AtMYB57/79 (weaker than 2 × Ma). (B) Overexpression of AtMYB2/57/79 in protoplasts. (C) Genes activated by AtMYB2 enrich its motifs in promoters. Almost all the AtMYB2 motifs enrich in the promoters of up-regulated DEGs, but not in promoters of the non-DEGs. (D) Genes regulated by ER0/1s are up-regulated by MYB2 overexpression. Genes harboring ER0/1s in promoters are overlapped with genes up-regulated by AtMYB2 OE. The significance of the overlap is from Fisher’s exact test. (E) Differentially expressed genes (DEGs) after AtMYB2 overexpression. AT2G16760 is the gene with the highest (and reliable, FDR < 1e-05) increase of expression after overexpression of AtMYB2, and contains an ER1a motif in its promoter. (F) AT1G76550 is regulated specifically by AtMYB2 through ER1a. The coverage tracks of RNA-seq and DAP-seq near the ER1a target gene AT1G76550. The ER1a CRE is indicated in grey. (G) Enrichment of AtMYB2 motifs in SELEX, DAP-seq, and promoters of up-regulated DEGs. Compared to DAP-seq (right), the enrichment of AtMYB2 motifs in SELEX (left) better correlates with their ability to activate transcription (x-axis: enrichment in promoters of up-regulated DEGs from RNA-seq). (H) ER0/1-specific CREs are up-regulated by OE of AtMYB2 but not AtMYB57/79. ER0/1-specific CREs (within AtMYB2 DAP peaks and < 500 bp from TSSs) are not bound by AtMYB57/79 (upper-left panel). Accordingly, the genes regulated by the ER0/1-specific CREs are significantly up-regulated by AtMYB2 but not AtMYB57/79 (lower-left panel and right panel). *** *p* < 0.001.


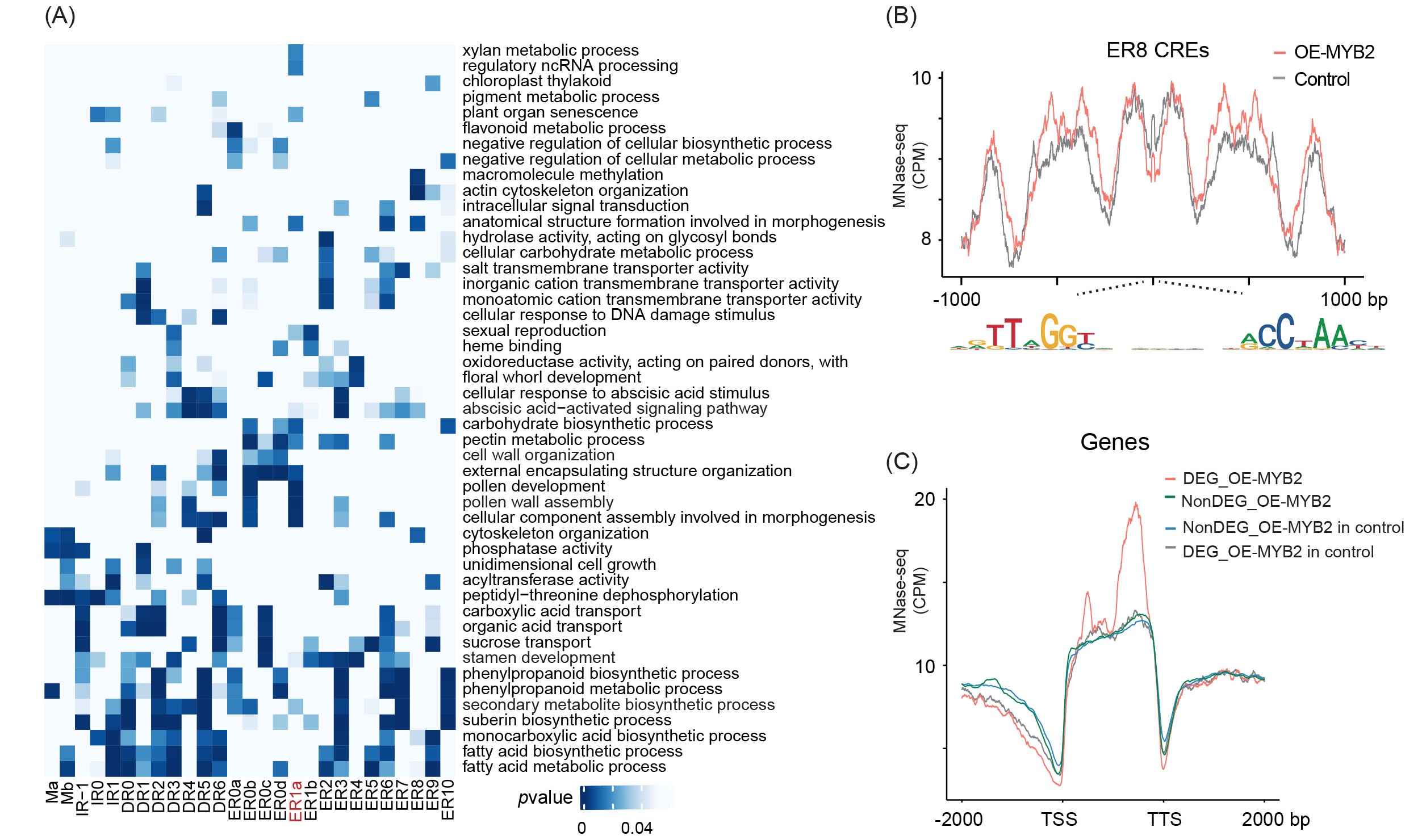


**Figure S7. Transcriptional regulation by AtMYB2.** (A) GO enrichments of genes up-regulated by different dimeric modes after AtMYB2 overexpression. (B) AtMYB2 binding is compatible with nucleosome occupancy. Nucleosome occupancy before and after overexpression of AtMYB2 in *Arabidopsis* protoplasts was measured by MNase-seq. AtMYB2 has little effect on nucleosome occupancy before (grey line) and after (red line) overexpression near its ER8 CREs. (C) DEGs induced by AtMYB2 show increased nucleosome occupancy near TTSs. MNase coverages near different gene sets are visualized. Note that the DEGs induced by AtMYB2 overexpression have increased nucleosome occupancy near the TTS region (red line). Such increased occupancy is not observed for non-DEGs after AtMYB2 overexpression (green line), and also not observed for both the DEGs and non-DEGs (the control lines) in the protoplasts before overexpression.

**REFERENCES**

1. Chow, ChiNga, TzongYi Lee, YuCheng Hung, GuanZhen Li, Tseng KuanChieh, YaHsin Liu, PoLi Kuo, et al. 2019. “PlantPAN3.0: a new and updated resource for reconstructing transcriptional regulatory networks from ChIP-seq experiments in plants.” *Nucleic Acids Research* 47:D1155–D1163. <https://doi.org/10.1093/nar/gky1081>

2. Jin, Jinpu, Feng Tian, DeChang Yang, YuQi Meng, Lei Kong, Jingchu Luo, and Ge Gao. 2017. “PlantTFDB 4.0: toward a central hub for transcription factors and regulatory interactions in plants.” *Nucleic Acids Research* 45:D1040–D1045. <https://doi.org/10.1093/nar/gkw982>

3. Heinz, Sven, Christopher Benner, Nathanael Spann, Eric Bertolino, Yin C. Lin, Peter Laslo, Jason X. Cheng, et al. 2010. “Simple combinations of lineage-determining transcription factors prime cis-regulatory elements required for macrophage and B cell identities.” *Molecular Cell* 38:576–589. <https://doi.org/10.1016/j.molcel.2010.05.004>

4. Wang, Baihui, Qiang Luo, Yingping Li, Liufan Yin, Nana Zhou, Xiangnan Li, Jianhua Gan, et al. 2020. “Structural insights into target DNA recognition by R2R3-MYB transcription factors.” *Nucleic Acids Research* 48:460–471. <https://doi.org/10.1093/nar/gkz1081>

5. Dm, Emms, and Kelly Steven. 2019. “OrthoFinder: phylogenetic orthology inference for comparative genomics.” *Genome Biology* 20. <https://doi.org/10.1186/s13059-019-1832-y>

6. Erijman, Ariel, Lukasz Kozlowski, Salma Sohrabi-Jahromi, James Fishburn, Linda Warfield, Jacob Schreiber, William S. Noble, et al. 2020. “A High-Throughput Screen for Transcription Activation Domains Reveals Their Sequence Features and Permits Prediction by Deep Learning.” *Molecular Cell* 78:890-902.e6. <https://doi.org/10.1016/j.molcel.2020.04.020>

7. Morffy, Nicholas, Lisa Van Den Broeck, Caelan Miller, Ryan J. Emenecker, John A. Bryant, Tyler M. Lee, Katelyn Sageman-Furnas, et al. 2024. “Identification of plant transcriptional activation domains.” *Nature* 632:166–173. <https://doi.org/10.1038/s41586-024-07707-3>


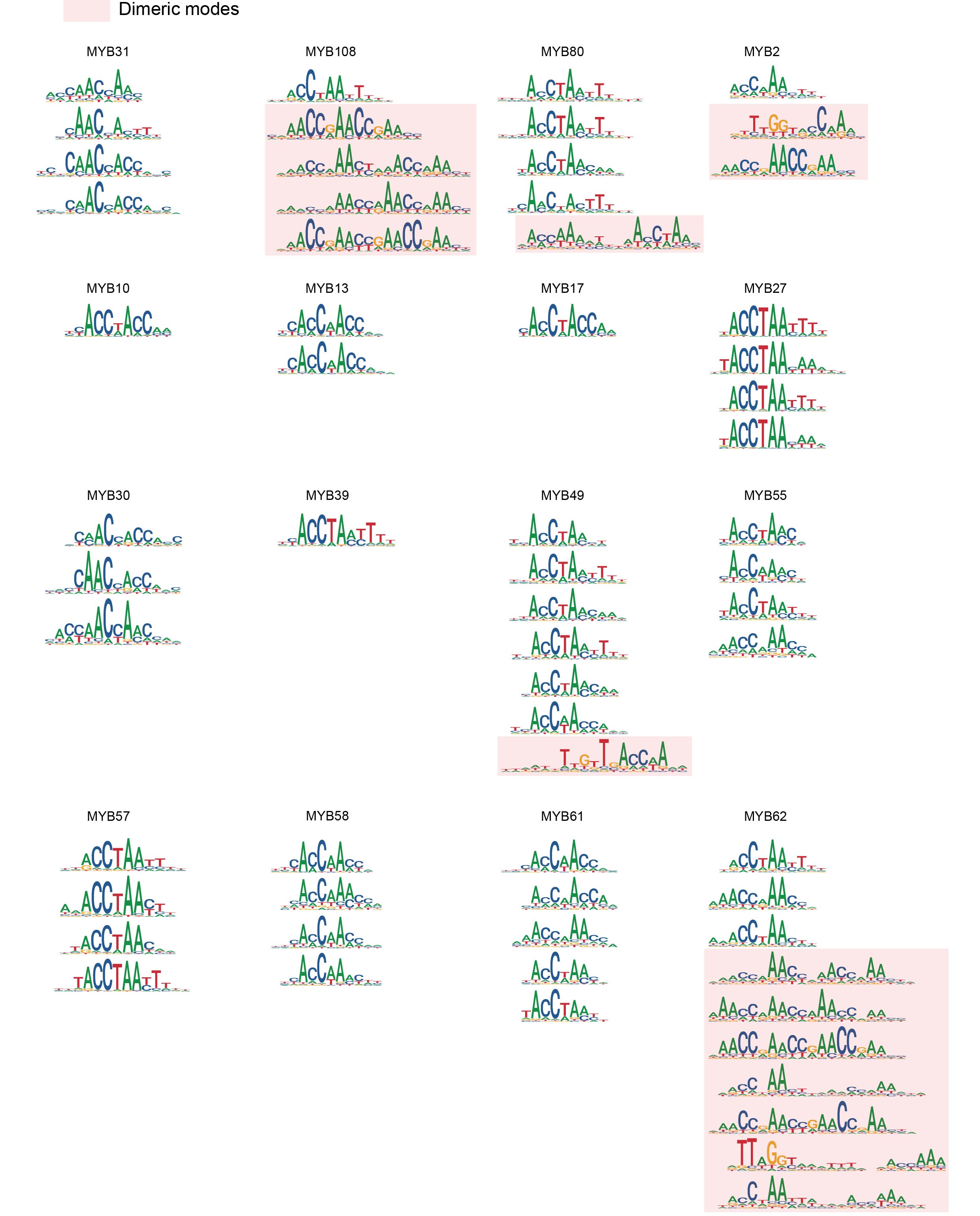


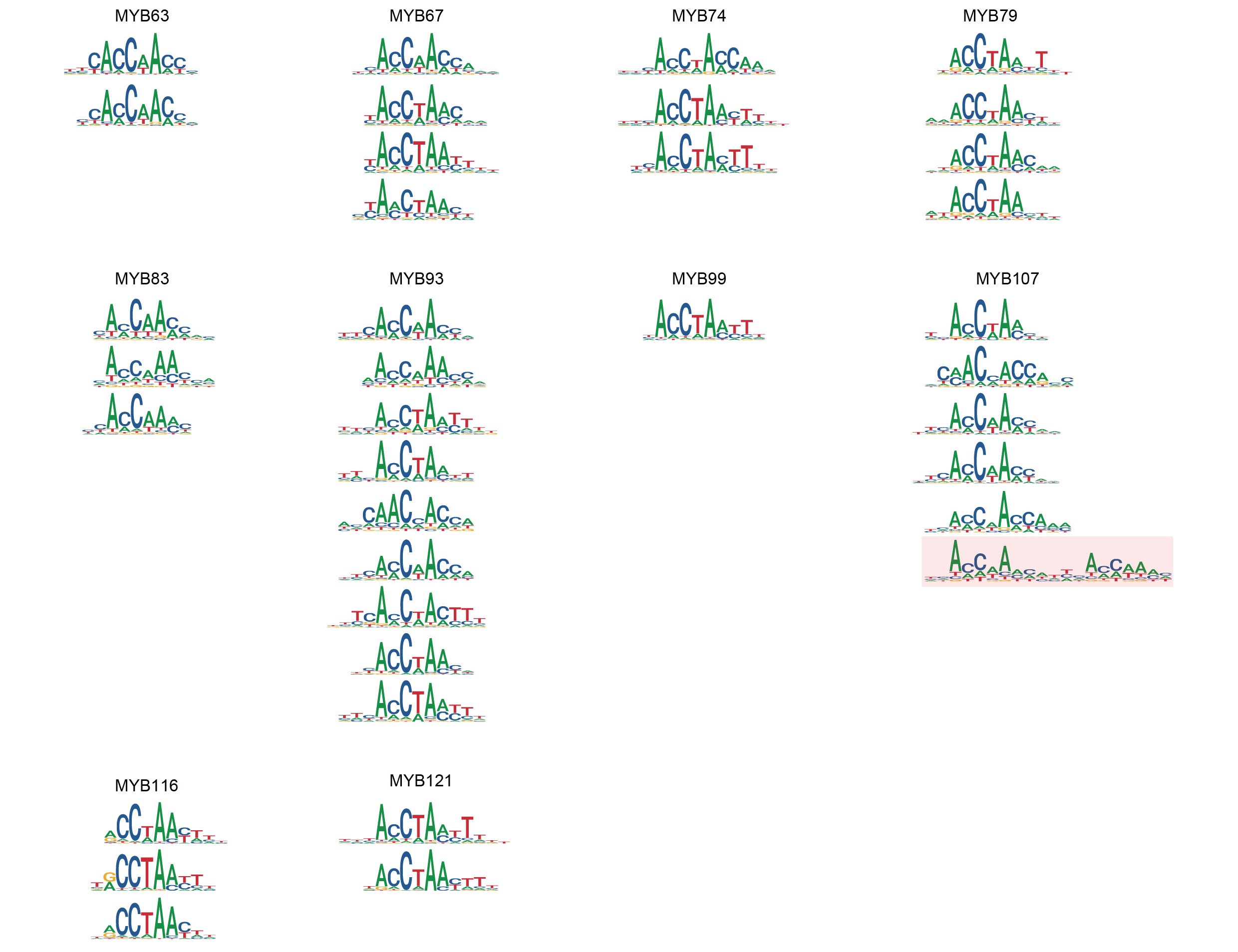


**Data S1 *De novo* motifs from published DAP-seq libraries of VIII AtMYBs (with Autoseed).** Autoseed was used to analyze DAP-seq peaks with the same parameters as SELEX analysis to examine whether DAP-seq libraries could also identify the dimeric binding modes.


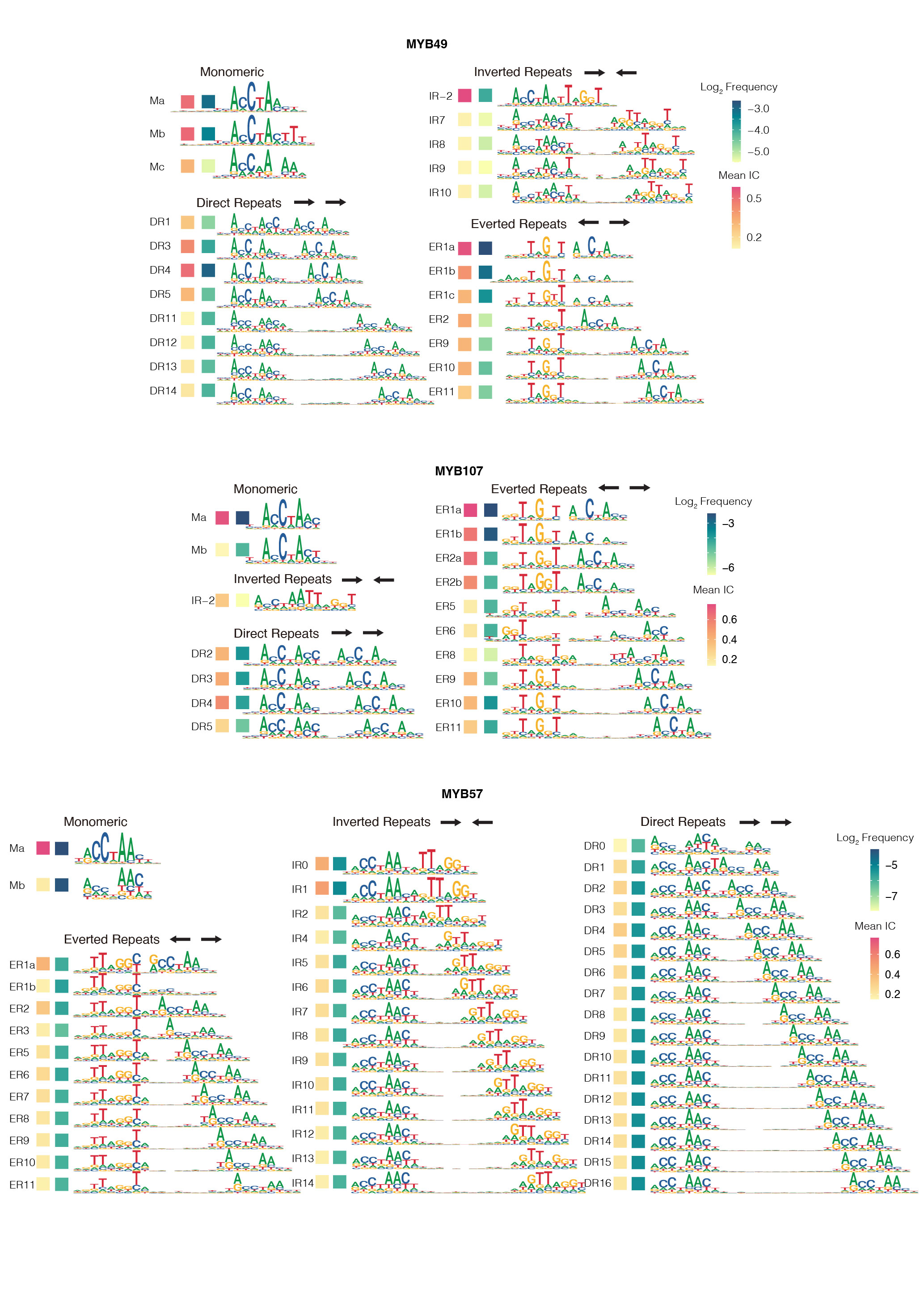

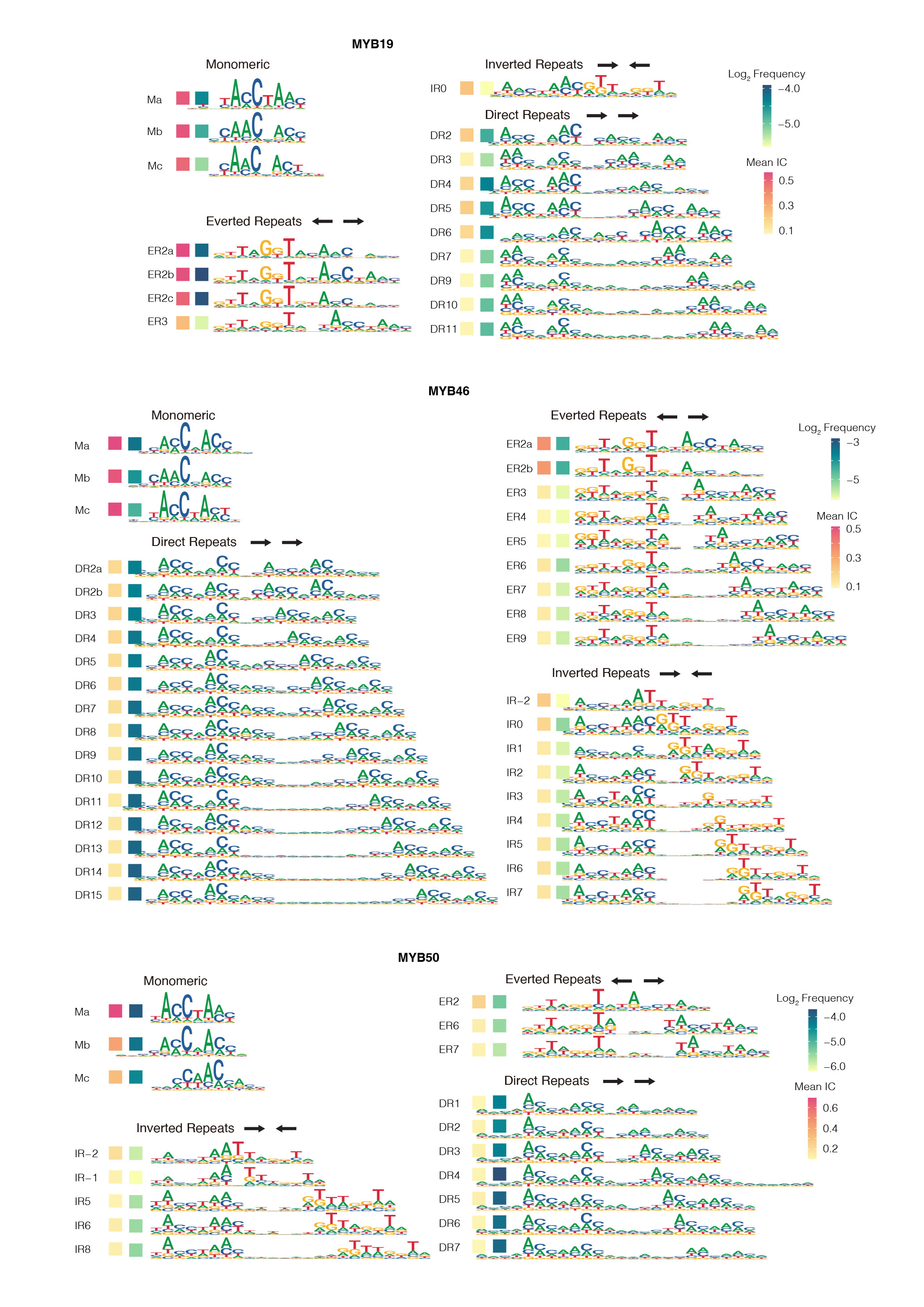

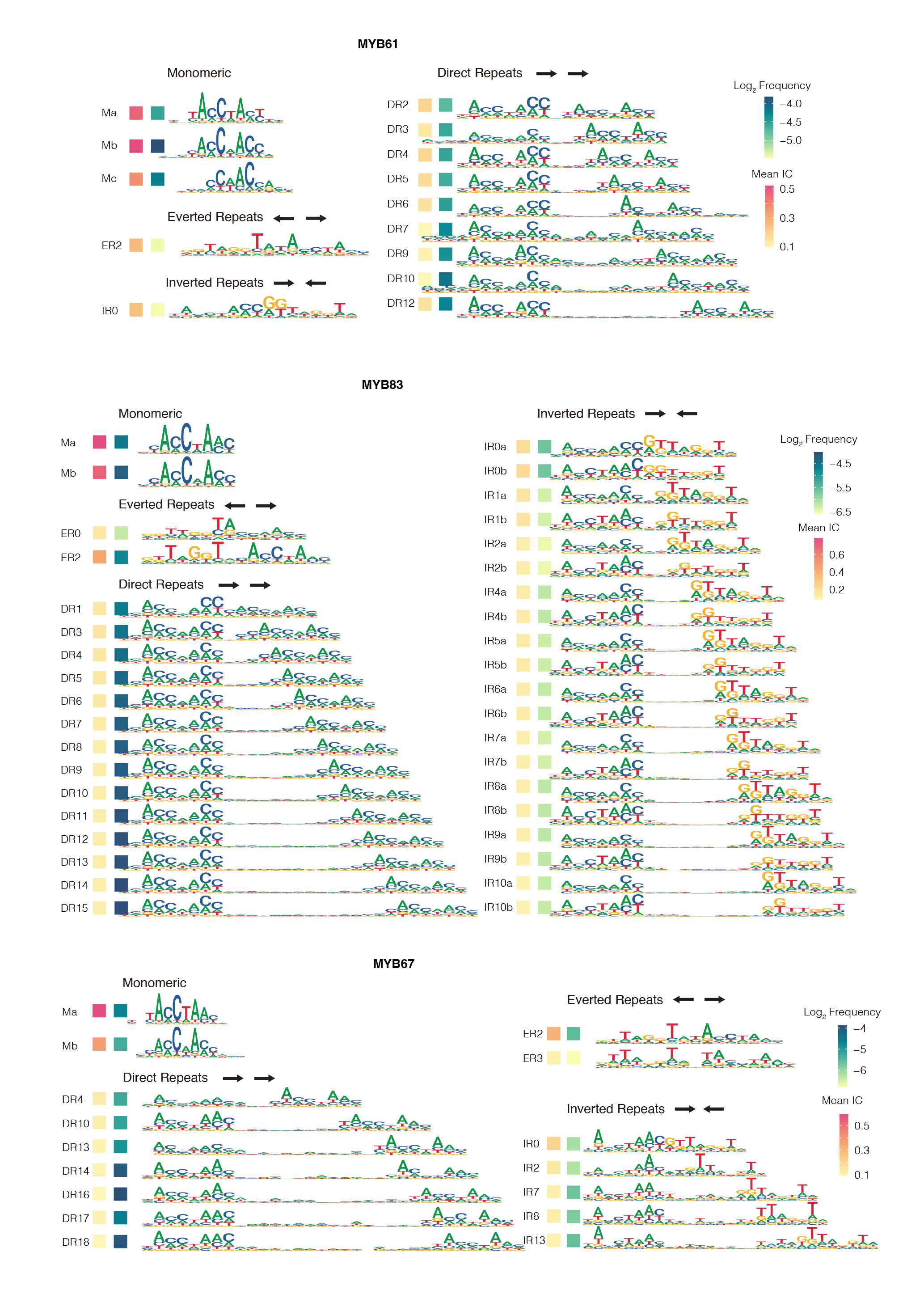

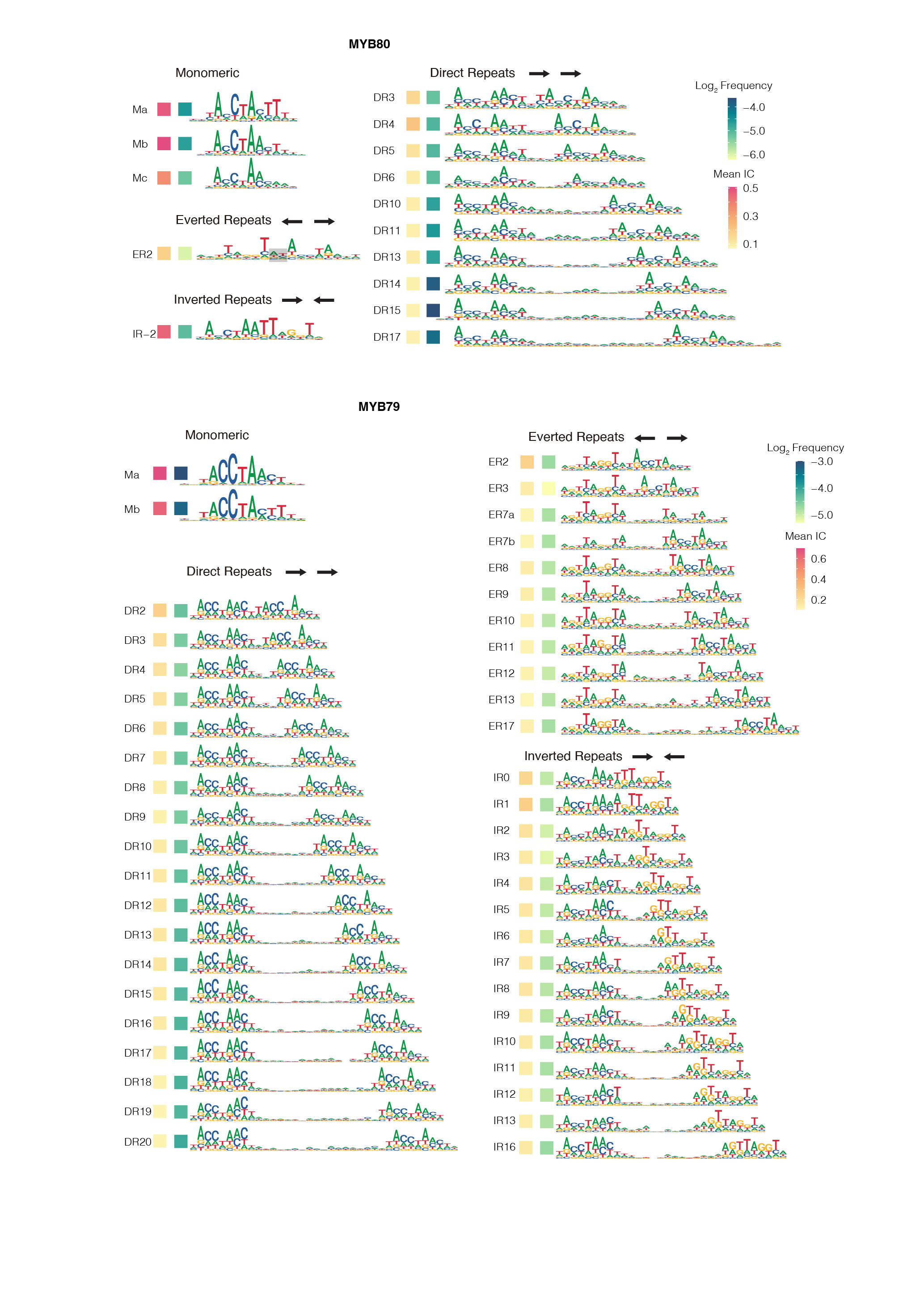

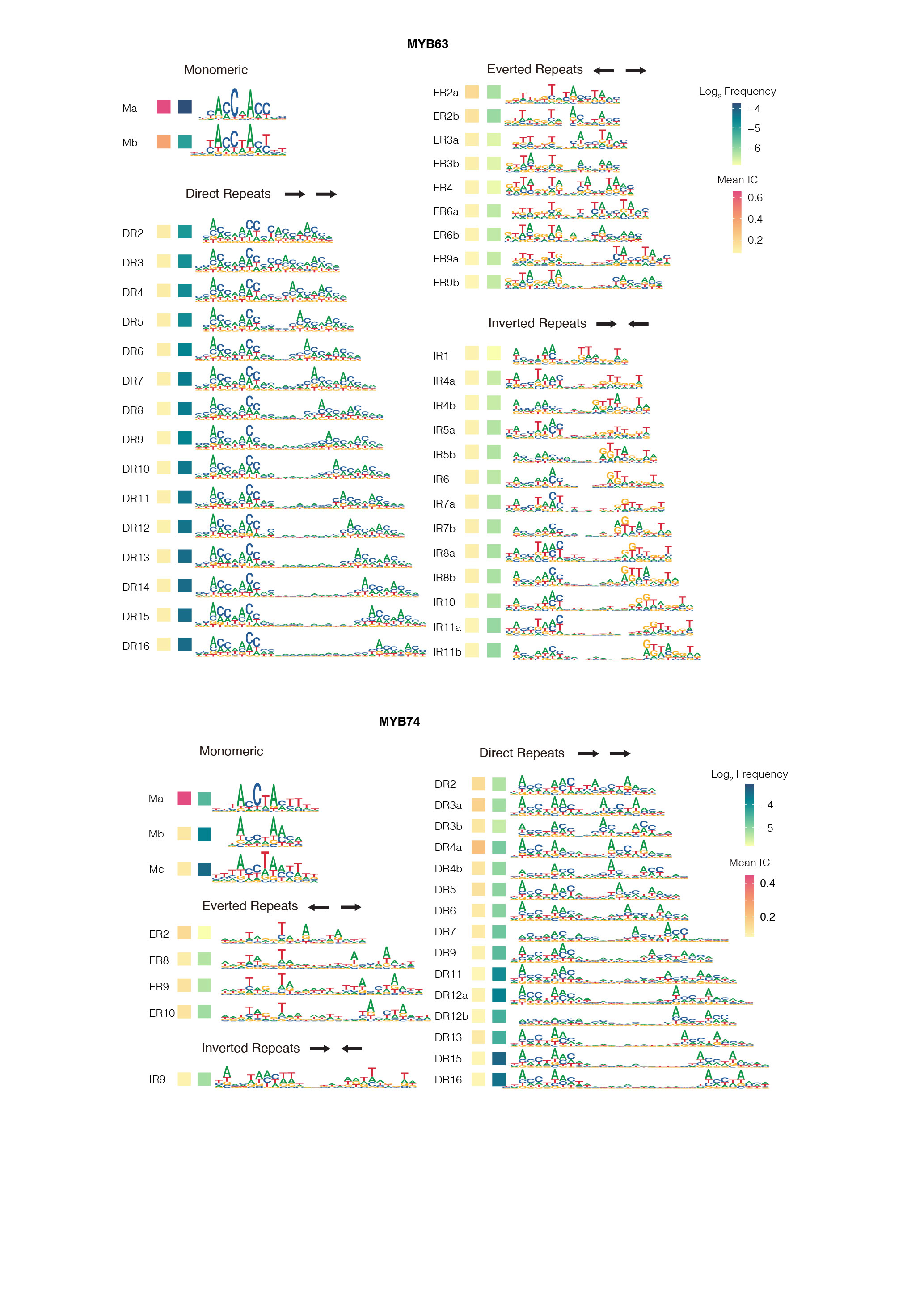

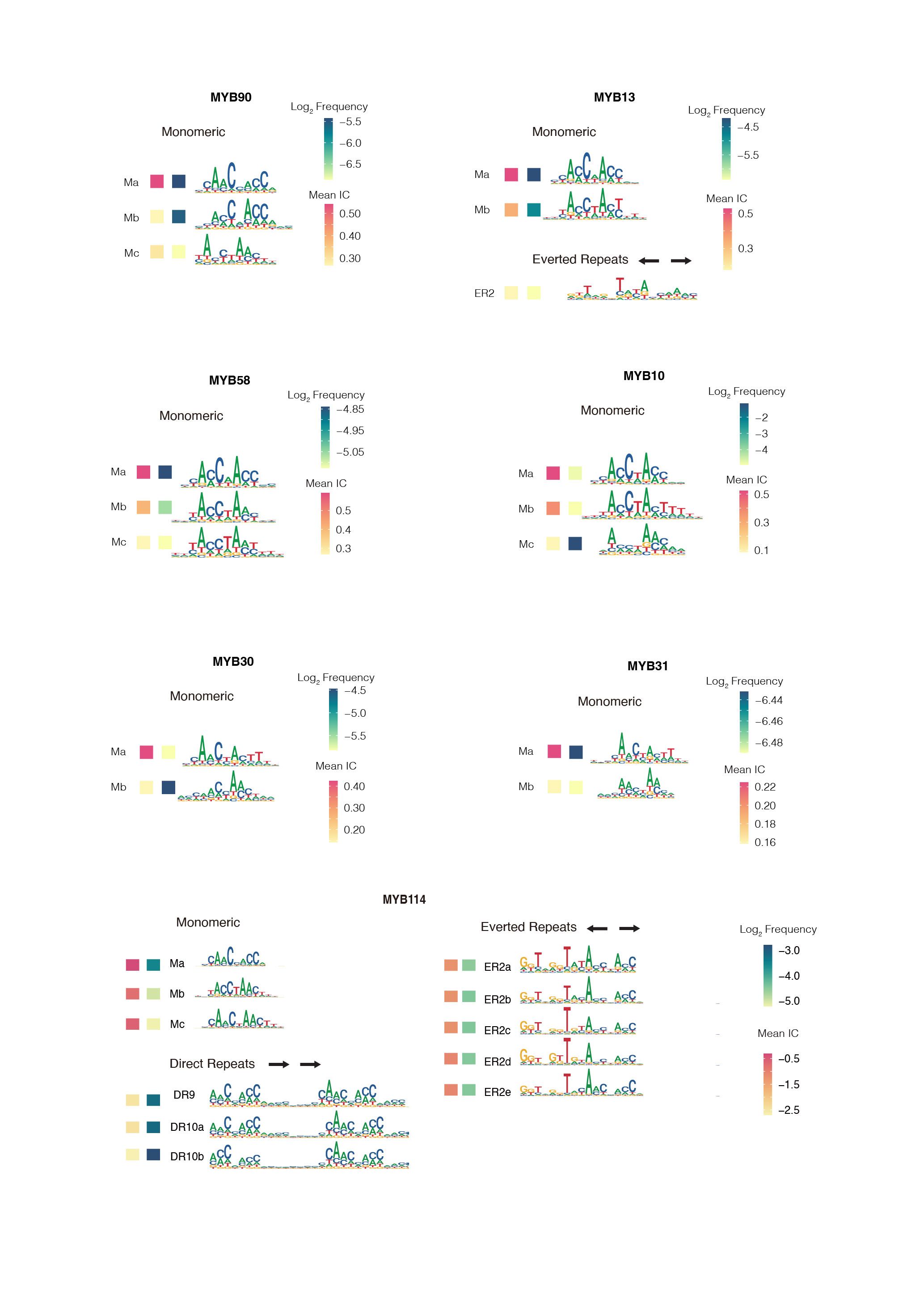

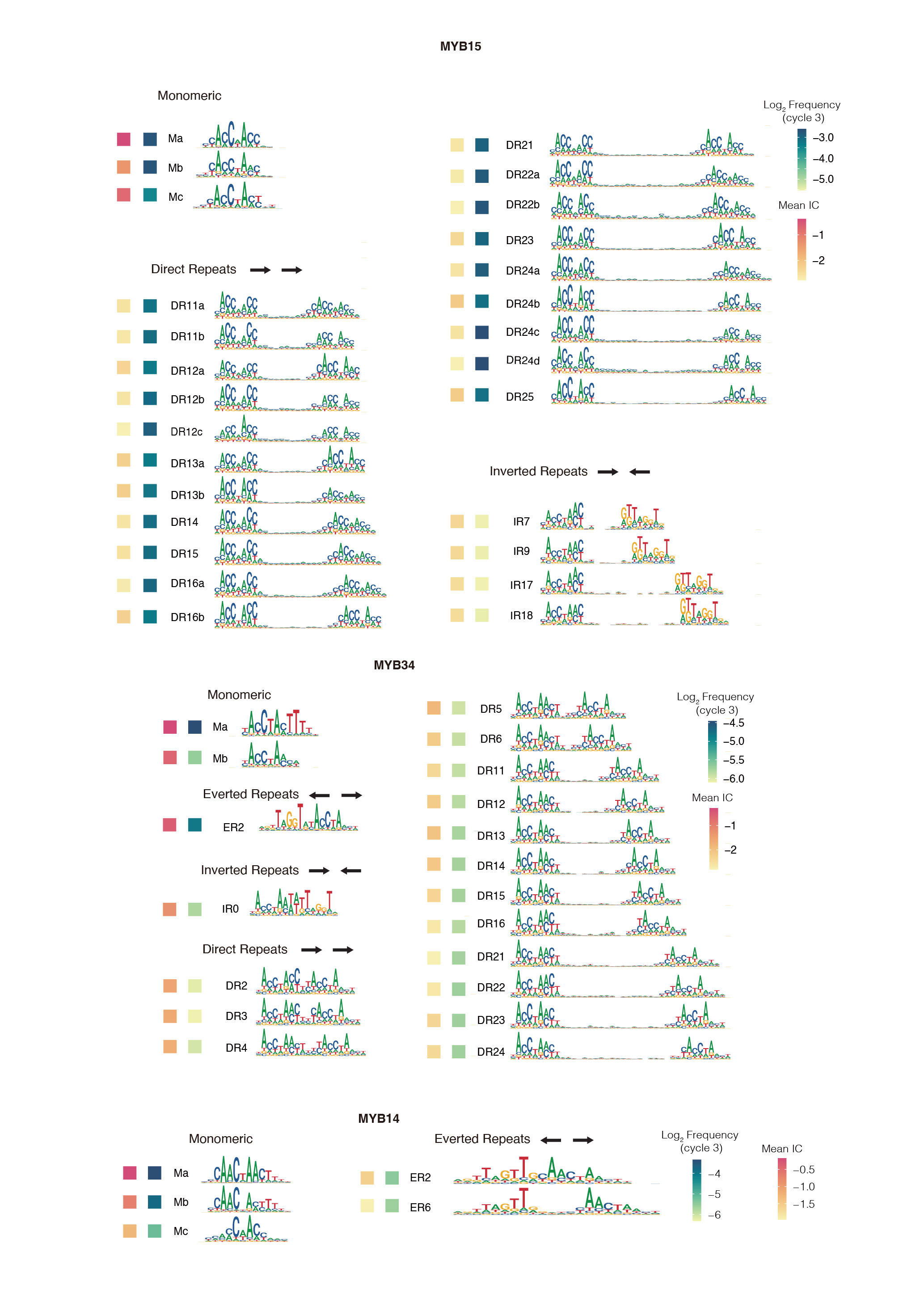

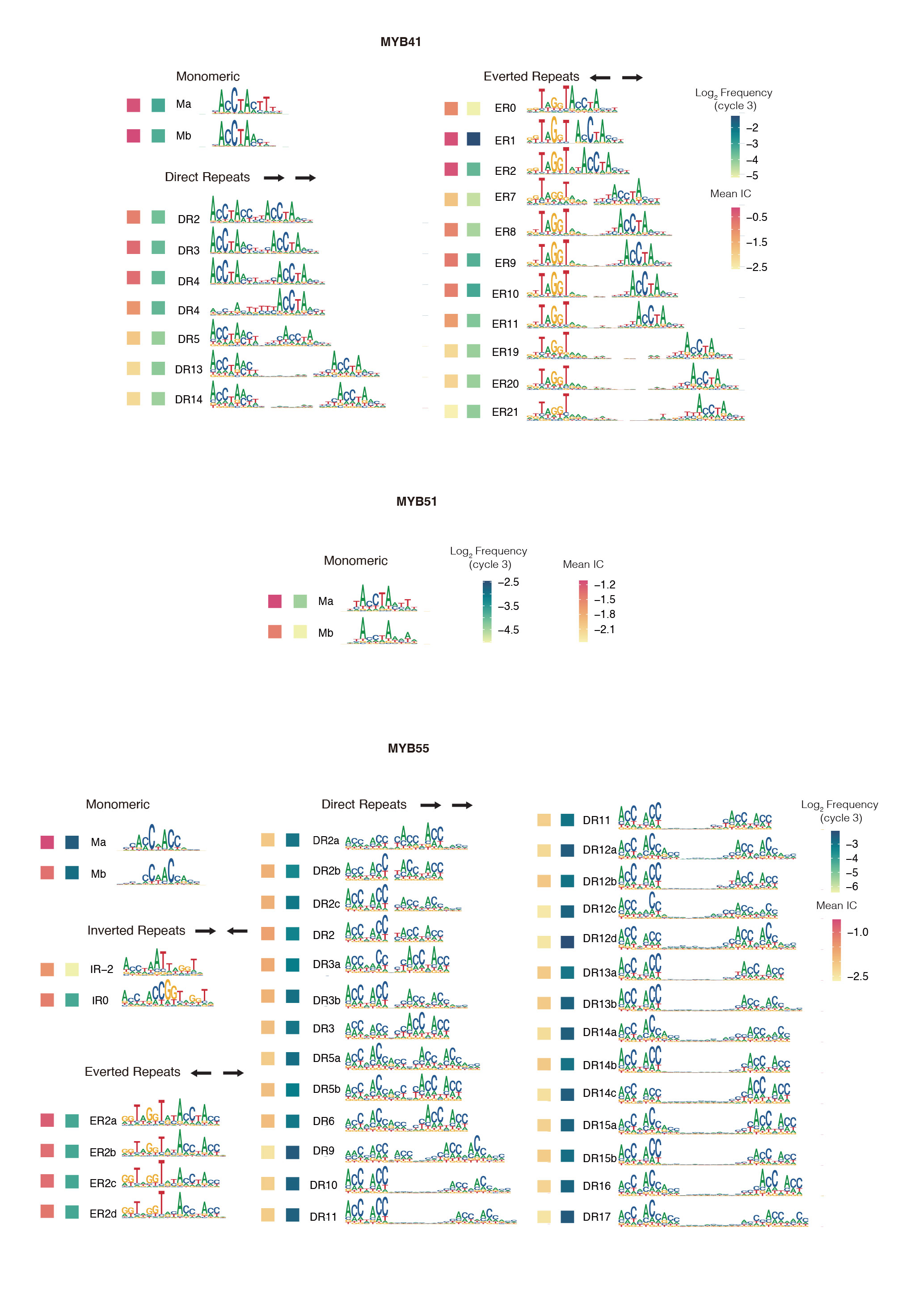

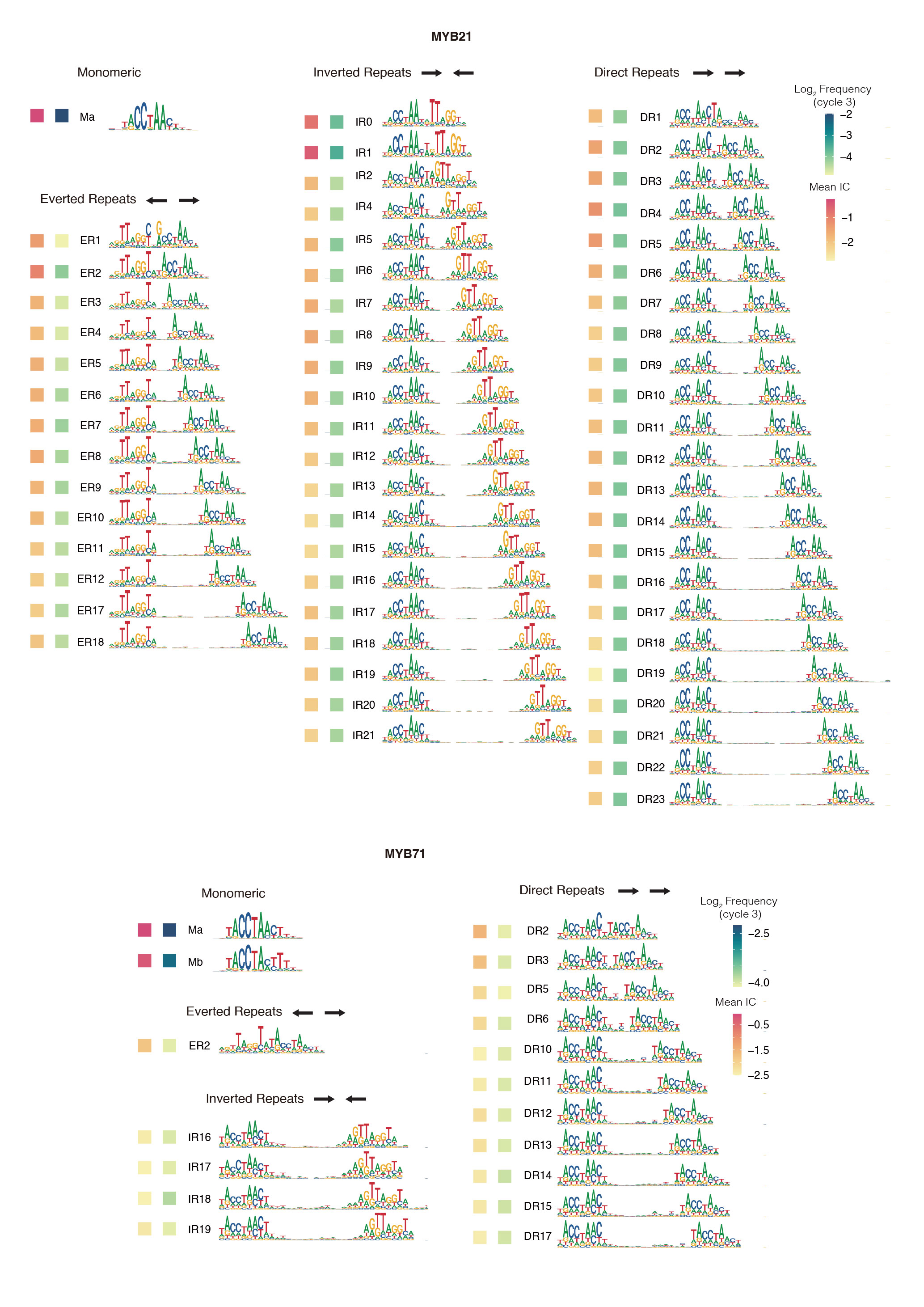

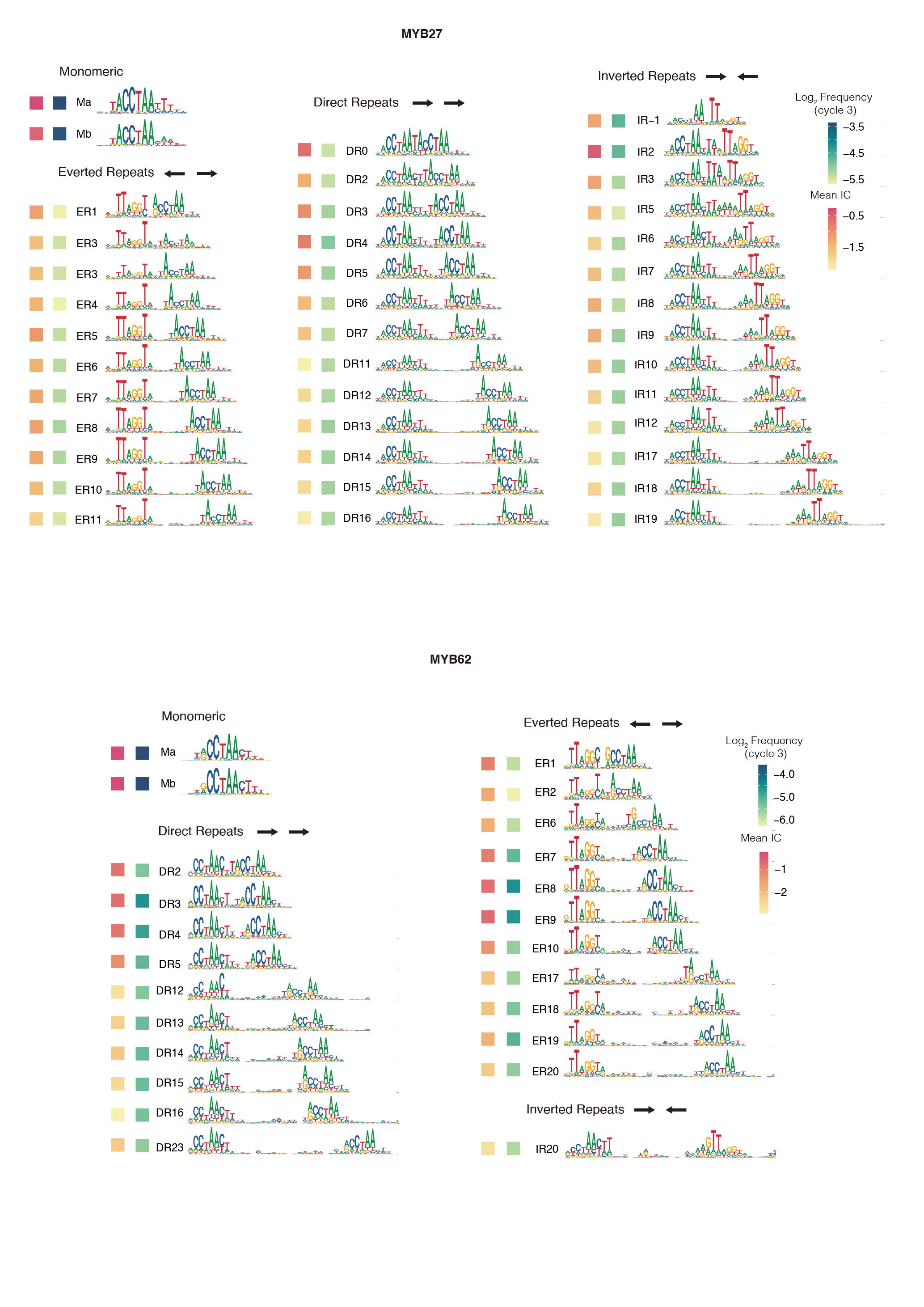

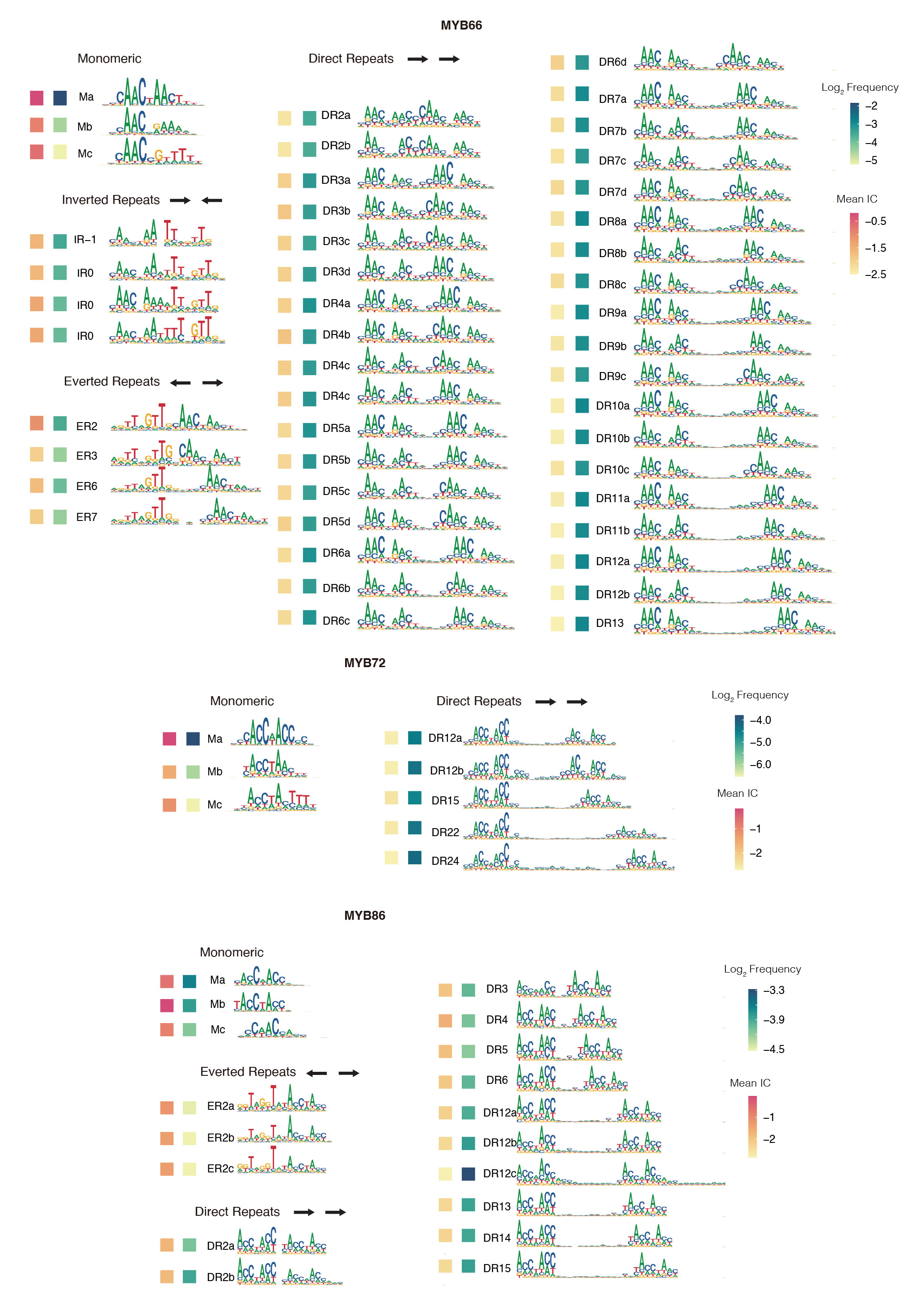

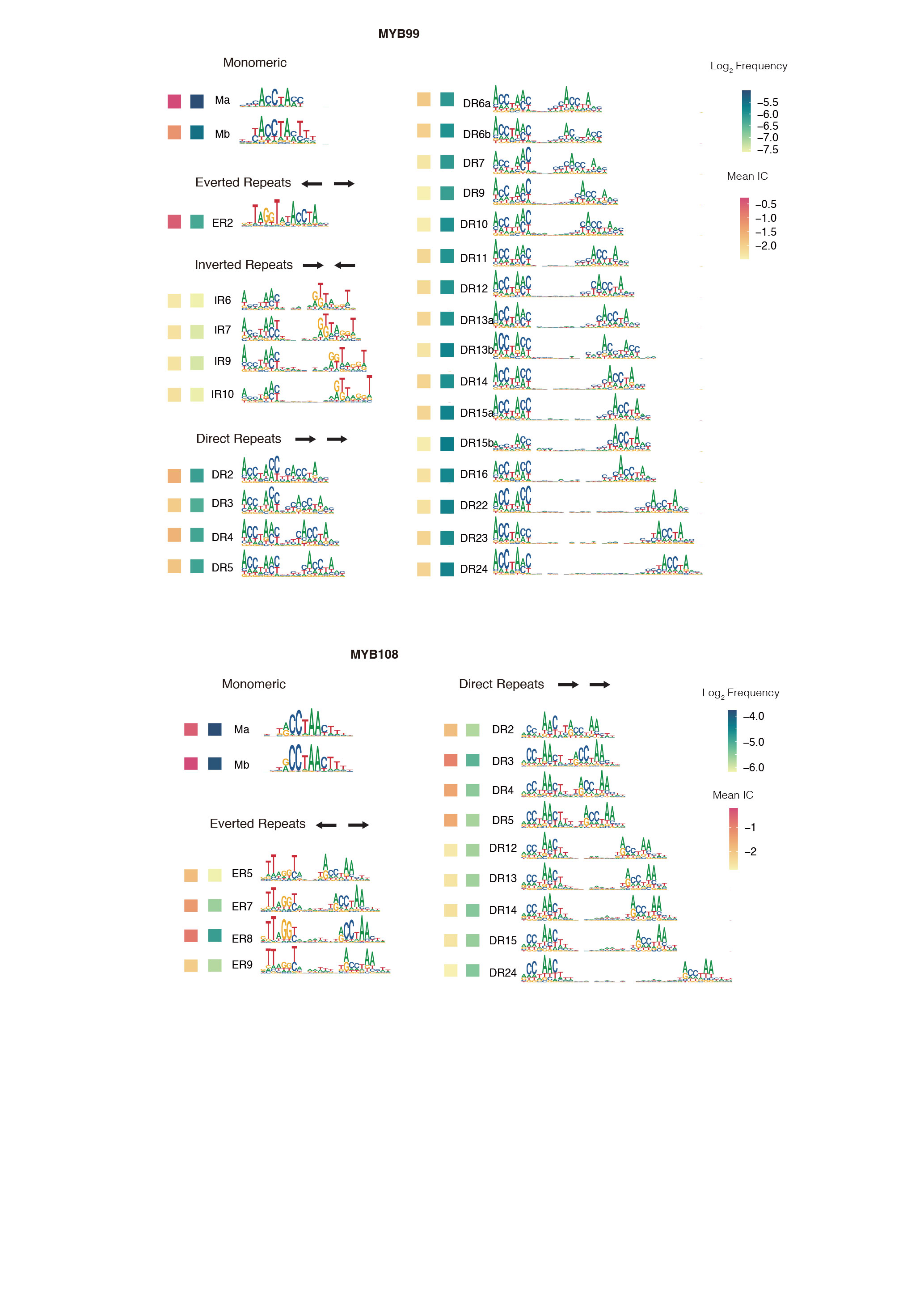

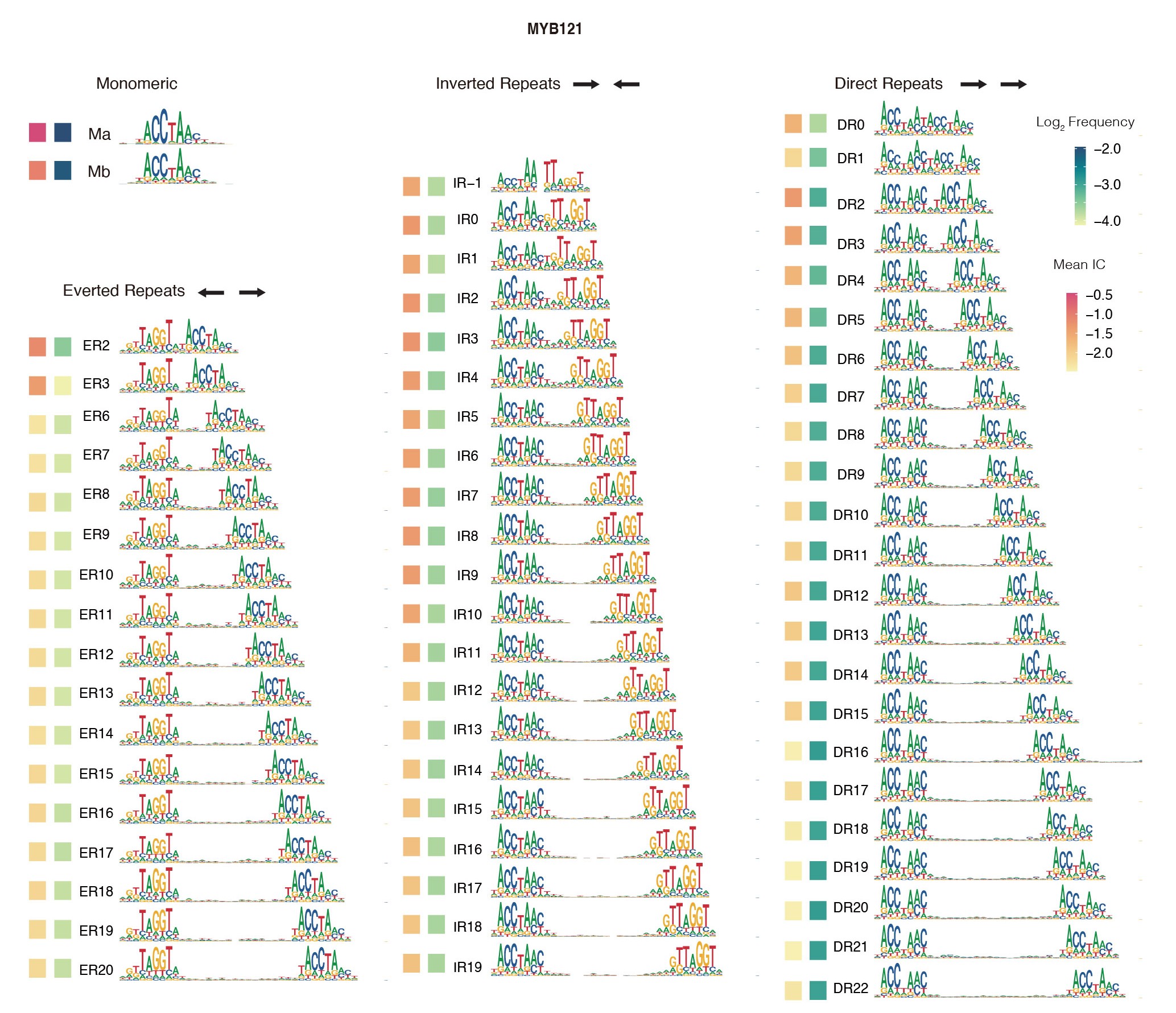


**Data S2 Monomeric and homodimeric motifs of VIII R2R3-AtMYBs (with Autoseed).** *De novo* discovered motifs in 38 R2R3-AtMYBs SELEX libraries, with their information content (red squares) and enrichment (green squares) indicated to the left. Dimeric motifs were named according to their relative orientations and spacings. In total, we identified 833 motif models.
